# An accurate, reliable, and universal qPCR method to identify homozygous single insert T-DNA with the example of transgenic rice

**DOI:** 10.3389/fpls.2023.1221790

**Published:** 2023-10-10

**Authors:** Hai Thanh Tran, Carly Schramm, My-my Huynh, Yuri Shavrukov, James C. R. Stangoulis, Colin L. D. Jenkins, Peter A. Anderson

**Affiliations:** College of Science and Engineering, Flinders University, Adelaide, SA, Australia

**Keywords:** quantitative real-time PCR, zygosity identification, homozygous plant, *NOS* terminator, *OsSBE4* reference gene, single T-DNA, copy number, transgenic rice

## Abstract

Early determination of transgenic plants that are homozygous for a single locus T-DNA insert is highly desirable in most fundamental and applied transgenic research. This study aimed to build on an accurate, rapid, and reliable quantitative real-time PCR (qPCR) method to fast-track the development of multiple homozygous transgenic rice lines in the T_1_ generation, with low copy number to single T-DNA insert for further analyses. Here, a well-established qPCR protocol, based on the *OsSBE4* reference gene and the *nos* terminator, was optimized in the transgenic Japonica rice cultivar Nipponbare, to distinguish homozygous single-insert plants with 100% accuracy. This method was successfully adapted to transgenic Indica rice plants carrying three different T-DNAs, without any modifications to quickly develop homozygous rice plants in the T_1_ generation. The accuracy of this qPCR method when applied to transgenic Indica rice approached 100% in 12 putative transgenic lines. Moreover, this protocol also successfully detected homozygous single-locus T-DNA transgenic rice plants with two-transgene T-DNAs, a feature likely to become more popular in future transgenic research. The assay was developed utilizing universal primers targeting common sequence elements of gene cassettes (the *nos* terminator). This assay could therefore be applied to other transgenic plants carrying the *nos* terminator. All procedures described here use standardized qPCR reaction conditions and relatively inexpensive dyes, such as SYBR Green, thus the qPCR method could be cost-effective and suitable for lower budget laboratories that are involved in rice transgenic research.

## Introduction

An efficient and productive protocol to identify homozygous plants plays a crucial role in molecular genetics and physiological studies, delivery of CRISPR-Cas9 cassettes for genome editing, and for molecular breeding of transgenic plants such as rice. A successful transformation process produces a first generation (T_0_) of hemizygous plants, where the T-DNA is inserted without an allelic counterpart. The T_0_ plants may contain one, two or multiple copies of the T-DNA in the host genome at the same or different locations. After self-pollination, the T-DNA segregates according to Mendelian principles. This means that the progeny in the subsequent generation (T_1_) are either homozygous, hemizygous or null for a particular T-DNA insert. To ensure stable inheritance of the T-DNA and any ongoing genetic or physiological analysis, only homozygous plants should be used, and preferably those with a single T-DNA insert. Thus, development of homozygous transgenic lines is a fundamental requirement in most of the downstream applications and studies of transgenic organisms, including plants. Therefore, an efficient and rapid technique for determining the T-DNA copy number, and screening for homozygous plants in the T_1_ generation, would fast-track this selection process.

To date, Southern blot and quantitative real-time PCR (qPCR) are the two most common methods for quantifying T-DNA copy number in transgenic plants. Southern blot analysis (Southern, 1975) is a powerful and reliable method but requires a large amount of genomic DNA and is time-consuming, in some cases hazardous and laborious, and thus is expensive. Especially for rice and other cereal crops which have a long-life cycle (4-5 months), large amount of space and resources are required for growth to generate subsequent generations. An alternatively to Southern blot analysis is qPCR, a rapid technique that is based on the detection of fluorescence generated during the amplification process (German et al., 2003; Bubner and Baldwin, 2004; Bubner et al., 2004). The fluorescence can be produced by an intercalating dye that fluoresces as it binds to double-stranded DNA or using a probe containing both a fluorophore and a quencher (TaqMan) targeted to an internal region of the transgene. During the extension step in the latter qPCR assay, DNA polymerase degrades the probe, releasing the fluorophore from the quencher and giving rise to fluorescence. The result of the PCR reaction is expressed as a cycle threshold (Ct) value. Other more technically sophisticated techniques have been developed from the basic TaqMan concept, but TaqMan requires relatively expensive probes (Baric et al., 2006) and it is less suitable for large-scale and universal testing applications. Droplet digital PCR (ddPCR) was evaluated and used to estimate T-DNA copy number in several crop species (Collier et al., 2017; Giraldo et al., 2019; Cai et al., 2020; Cai et al., 2021), homozygous transgenic tobacco (Głowacka et al., 2016), and maize (Xu et al., 2016). Again, ddPCR replies on an initial restriction digestion step, expensive probes and more expensive droplet digital PCR systems to estimate T-DNA copy number, and this thus is less suited to lower budget laboratories.

An alternative qPCR-based method known as the comparative Ct (2^-ΔΔCt^) method is reported for determining copy number and/or zygosity of transgenes (Bubner and Baldwin, 2004). Many analyses on copy number and/or zygosity of transgenes have employed the qPCR technique in several species such as maize (Schmidt and Parrott, 2001; Song et al., 2002; Shou et al., 2004; Xu et al., 2016), tomato (Mason et al., 2002; German et al., 2003), tobacco (Bubner et al., 2004), wheat (Li et al., 2004; Gadaleta et al., 2011; Giancaspro et al., 2017), and rice (Yang et al., 2005). Most studies showed a robust effectiveness of copy number determination, but effective outcomes for zygosity were unclear, except for the studies of Ingham et al. (2001). Later, it was shown that qPCR could be used to screen for homozygous transgenic cereal crops (Mieog et al., 2013; Wang et al., 2015) but the previous protocols were not universally adaptable for different T-DNAs and they were not consistent in all plants with identical zygosity. In most transgenic plant breeding studies, a large number of transgenic lines transformed with different gene expression cassettes can be generated. Therefore, a universal and fast-tracking method of homozygous plant identification would be advantageous. Here, the universal qPCR assay was optimized for accurate and reliable determination of zygosity using the example of transgenic rice plants. Compared to previous methods, this standardized qPCR assay is cost-effective and useful as a high-throughput method for fast-tracking identification of homozygous plants carrying single or low insert numbers in a single generation. It was successfully adopted to identify many homozygous single- or two-insert T-DNA rice plants in the T_1_ generation carrying four different gene expression cassettes, all with the same *nos* terminator sequence, without any modification.

## Materials and methods

### *Agrobacterium*-mediated transformation and transgenic plants

**Figure 1:**
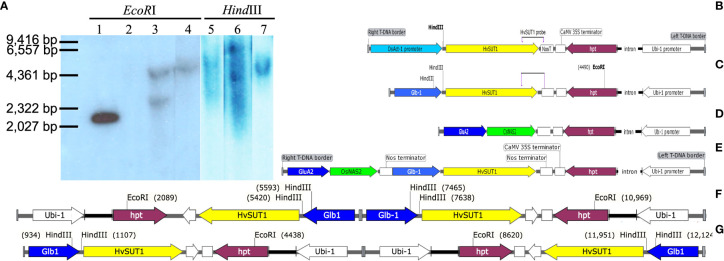
Southern blot analyses of the transgenic lines and schematic representation of the four T-DNAs used for rice transformation. **(A)** Genomic DNA of the T_0_ transgenic lines was digested with *Hind*III or *Eco*RI, then probed with a fragment of the *HvSUT1* gene. Migration of *Hin*dIII-digested lambda marker is noted. Lane 1 contains 100 pg of the pIPKb001 plasmid (EU161567) carrying *Glb : HvSUT1:NosT* used as a positive control. Lane 2 is the genomic DNA of non-transgenic rice Nipponbare digested with *Eco*RI used as a negative control. Lane 3 and 5 showed two bands in the genomic DNA of the G1.4 transgenic line carrying the *Glb : HvSUT1:NosT* digested by *Eco*RI and a single band by *Hind*III, respectively. Lane 4 and 6 showed a single band in the genomic DNA of the G3.1 transgenic line carrying the *Glb : HvSUT1:NosT* digested by *Eco*RI and two bands by *Hind*III, respectively. Lane 7 showed a single band in the DNA genomic of the A5.1 transgenic line carrying the *Act-1:HvSUT1:NosT* digested by *Hind*III. The *Act-1:HvSUT1:NosT* construct with one *nos* terminator **(B)** and the T-DNA of the *Glb-1:HvSUT1:NosT* with one *nos* terminator **(C)** with the *Hind*III and *Eco*RI sites and a position of the probe of *HvSUT1*. The T-DNA containing two stacked genes with two *nos* terminator regions (the *Glb-1*:*HvSUT1:NosT* and *GluA2*:*OsNAS2:NosT)* **(D)** and the T-DNA of *GluA2*:*OsNAS2:NosT* with one *nos* terminator **(E)**. The T-DNA of the G3.1 transgenic line **(F)** and the G1.4 transgenic line **(G)** with the insertion of two T-DNAs in a tandem repeat, but inverted orientation. *Glb-1* and *GluA2*: rice endosperm specific promoter *Globulin 1* and *Glutelin A2*; *Act-1*: the rice actin 1 gene promoter; *HvSUT1*: barley sucrose transporter 1, *OsNAS2*: rice nicotianamine synthetase 2; hygromycin phosphotransferase (*hpt*) gene under control of the *Ubi-1* promoter and the *CaMV 35S* terminator (35S T). Illustrations of the DNAs were prepared using SnapGene (GSL Biotech).

Multiple Japonica and Indica transgenic rice plants were generated via *Agrobacterium*-mediated transformation with four different T-DNAs (**Figure 1**). The transgenic Japonica lines carrying the *Act-1:HvSUT1:NosT* and *Glb-1:HvSUT1:NosT* cassettes were abbreviated to A lines and G lines, respectively. The Indica rice lines containing the *Glb-1:HvSUT1:NosT*, *GluA2:OsNAS2:NosT*, and *GluA2:OsNAS2:NosT-Glb-1:HvSUT1:NosT* were designated as SC1, SC2 and DC1, respectively. All T-DNAs contained a single common sequence of the nopaline synthase (*nos*) terminator, except for the DC1 T-DNA which contained two *nos* terminators. Illustrations of the T-DNAs were prepared using SnapGene (GSL Biotech).

### Primer design

The rice genes coding for branching enzyme (*OsSBE4*) and sucrose phosphate synthase (*OsSPS*), are appropriate reference genes in the qPCR assay as they are each present as two copies at a single locus in the rice genome (Mizuno et al., 2001; Jiang et al., 2009). All oligonucleotide primers were designed using the Primer3 software (bioinfo.ut.ee/primer3-0.4.0/primer3/). Some parameters were modified from the default setting to achieve similar amplification efficiencies between the reference genes and T-DNAs in the qPCR assay, including product size (80-200 bp) and primer Tm (59-65 °C). The selected primer pairs were checked by BLASTn against representative genomes for *Oryza sativa* ssp. Japonica and Indica to check the specificity of each primer pair. Folding template of the reference genes and T-DNAs were analysed by OligoArchitect™ Primer (offered by Sigma-Merck, USA) to eliminate primers that matched with predicted secondary structures in the templates. UNAFold software (http://sg.idtdna.com/UNAFold) was used to predict whether amplicons generated any secondary structure at the annealing temperature. Primer pairs that passed all of these requirements were synthesized by Sigma-Merck (**Supplementary Table S1**).

### End-point PCR for confirmation of T-DNA integration

Rice genomic DNA (gDNA) used for end-point PCR and the qPCR assay was isolated from leaves (100 mg) of transgenic lines (T_0_, T_1_ or T_2_) using an Isolate II plant DNA kit (Bioline, USA). The gDNA was suspended in 50 μl of elution buffer (5 mM Tris-HCl, pH 8.5) and the DNA samples were diluted to achieve an average DNA concentration of around 4.26 ng/ul using sterilized MiliQ water. The quantity of gDNA was estimated using a Nanodrop 2000 instrument (Thermo Fisher Scientific, USA). For end-point PCR (**Supplementary Table S2**), a 25 µl reaction consisted of 1 × GoTaq Green Reaction Buffer (Promega, USA), 2 mM MgCl_2_, 0.4 mM dNTPs, 0.4 µM for each primer and 2 µl of 4.26 ng/µl DNA template. The PCR cycling protocol was as follows; initial denaturation for 10 min at 95 °C, followed by 30 cycles of 95 °C for 30 sec, 64 °C for 30 sec and 72 °C for 30 sec, ending with 10 min at 72 °C. PCR products were visualized on a 1.5% agarose gel.

### Standard curve establishment

An efficient, reproducible, and dynamic range qPCR assay was determined by making a standard curve using a 10-fold serial dilution of the target sequence (10^4^, 10^3^, 10^2^, 10^1^ and 10^0^ copies/μl). The absolute quantification of the copy number of the target sequence per haploid genome was calculated based on the mass of genomic DNA and the genomic DNA concentration determined by UV absorption at 260 nm based on the protocol of Applied Biosystems (Applied Biosystems, 2003). The mass of a single copy of the rice genome (haploid) was calculated as follows:


$$m=n\;\times \;(1.096\times 10^{-21})\;\text{g}/\text{bp,}$$

where m is mass (g) of genomic DNA and n is genome size (389 Mb) (Sequencing Project International Rice, 2005).

Then mass of the rice genomic DNA needed to achieve 10^4^ copies of the rice genome was calculated as follows


$$mass\;of\;gDNA\;needed=m\times 10^{4\;}\left(\text{g}\right).$$

For this case, 4.26 ng/μl was equivalent to 10^4^ copies/μl of the rice genome. The stock concentration of rice gDNA was determined by UV absorption at 260 nm and diluted to achieve 4.26 ng/μl (10^4^ copies/μl). The 10^4^ copies/μl was serially diluted 10-fold in sterile Milli Q water to reach 10^3^, 10^2^, 10^1^ and 10^0^ copies/μl of the rice gDNA.

The standard curve was generated from Ct values plotted against the logarithm of the template concentration at each dilution with three replicates. Amplification efficiency was calculated from the slope of the standard curve (Bio-Rad Laboratories, 2006). The amplification efficiencies of the primer pairs for the reference genes and the T-DNAs were then compared with each other and chosen when within 5% of each other, and as close to 100% as possible. Based on the standard deviation of five dilutions in the standard curve, a concentration of DNA template was chosen with the lowest standard deviation and then used in the qPCR assay for zygosity analysis.

### qPCR assay for zygosity identification

Reactions were done in a CFX96™ Real-time PCR detection instrument (BioRad, USA). A 10 μl reaction consisted of 5 μl (1 ×) KiCqStart SYBR Green qPCR ReadyMix (Sigma-Merck, USA), 300 nM for each primer and 2.5 μl of gDNA template (0.426 ng/μl ~10^3^ copies/μl). A standard two-step protocol was used as follows: 1 cycle for DNA denaturation at 95 °C for 10 min, followed by 40 cycles of 95 °C for 15 sec and 60 °C for 30 sec and a melting curve was generated in 0.5 °C increments starting at 60 °C. Reactions for both reference gene (*OsSBE4*) and the T-DNAs were run in triplicate.

As the amplification efficiency of the reference gene and T-DNAs were similar and nearly 100% and within 5% of each other, the zygosity of transgenic plants was determined by the Livak method, 2^-ΔΔCt^ (Livak and Schmittgen, 2001). The difference between the Ct values of the T-DNAs and reference gene for the test plant was calculated (ΔCt), then normalized to the ΔCt for a calibrator plant to obtain copy number calculation (ΔΔCt). The resulting ΔΔCt value was incorporated to determine zygosity of the test plant. The 2^-ΔΔCt^ method was calculated using the following steps.

First, the Ct values of the T-DNAs (for example *HvSUT1* and *Nos* terminator) were normalized to that of the reference gene (*OsSBE4*) for both test plants and calibrator:


ΔCt (test)=Ct (target, test)−Ct (ref,test)

ΔCt (calibrator)=Ct (target, calibrator)-Ct (ref, calibrator).

Second, the ΔCt of test plant was normalized to that of the calibrator to obtain ΔΔCt:


ΔΔCt=ΔCt (test)−ΔCt (calibrator).

Finally, the 2-fold difference was then found by 2 to the power of -ΔΔCt:


$$2^{-\text{ΔΔ}Ct}$$

If the calibrator was hemizygous (that is a single-insert T_0_ plant), the 2^-ΔΔCt^ of a homozygous single-insert plant will be twice that of the calibrator.

### Southern blot for determining insert number of T-DNAs

Southern blot analysis was conducted to provide a confirmation of T-DNA insert number independent to the qPCR assay. Rice gDNA of T_0_ transgenic Japonica lines was digested with *Hind*III or *Eco*RI (New England BioLabs, USA) in 30 μl reactions which consisted of 3 μl buffer (10 ×), restriction enzyme (30 units) and 3 μg gDNA. The reaction was incubated at 37 °C for at least 4 hrs. Digested fragments were separated on a 0.8% agarose gel by electrophoresis at 30-40 V overnight. After the separation of the DNA fragments was completed, the DNA was depurinated by incubating the gel in 0.25 M HCl for 10 min. The gel was rinsed in MilliQ water three times before the DNA was denatured in the high salt denaturation solution for 30 min. Afterward the DNA was transferred directly to Biodyne B nylon membrane (Pall Life Sciences, USA) by capillary transfer overnight in 20 × SSC buffer (pH 7) containing sodium chloride (3 M) and sodium citrate buffer (0.3 M). The membrane was then dried at 80 °C for 15 min, and then fixed with UV cross-linker (GS Gene linker UV chamber, BioRad, USA) at 150 mJoule for 10 sec. After fixation, the membrane was processed with hybridization and detection.

A 498-bp fragment amplified from the *HvSUT1* plasmid was used as a probe template for radioactive ^32^P-dCTP labelling using random primers (Geneworks, Australia), which consisted of 3 μl of random primer (40 μM), 5 μl of 20 ng/μl probe template, 12.5 μl of 2 × oligo labelling buffer, 1.5 μl of DNA polymerase (Klenow fragment) (2 units/μl) and 5 μl of ^32^P-dCTP in a 27 μl reaction. The labelled probe was incubated at 37 °C for 1-2 hrs, and then isolated by ethanol precipitation. Before hybridization, the membrane was mixed well and incubated with 5 × SSC overnight in the hybridization oven at 65 °C. The hybridization and detection processes were carried out according to Weiss (1992).

## Results

### A well-optimized qPCR protocol for homozygous single-insert determination

A transgenic Japonica rice line overexpressing the *Act-1:HvSUT1:NosT* (designated A5.1) was confirmed to carry a single insert in its genome by Southern blot, following digestion of genomic DNA with *Hind*III, which cuts once in the T-DNA construct (**Figures 1A, B**). The genomic DNA of this single copy line was used to optimize conditions of the qPCR protocol. For the expression construct *Glb-1:HvSUT1:NosT* (transformed plants designated G lines), there was a single *Eco*RI recognition site in hygromycin phosphotransferase (*hpt*) gene near on the left T-DNA border and two *Hind*III recognition sites close to the *HvSUT1* gene near the right border of the T-DNA construct. Two Southern blot analyses of the G lines were carried out with *Hind*III and *Eco*RI digested gDNA. The G1.4 line showed two bands in *Eco*RI digested DNA and a single band in *Hind*III digested DNA, while the G3.1 line had two hybridising fragments in *Eco*RI digested DNA and one fragment in the *Hind*III digestion (**Figure 1A**). The two G3.1 and G1.4 transgenic lines were predicted to contain two inserts of the T-DNA in a tandem repeat, but inverted orientation (**Figures 1F, G**).

**Figure 2:**
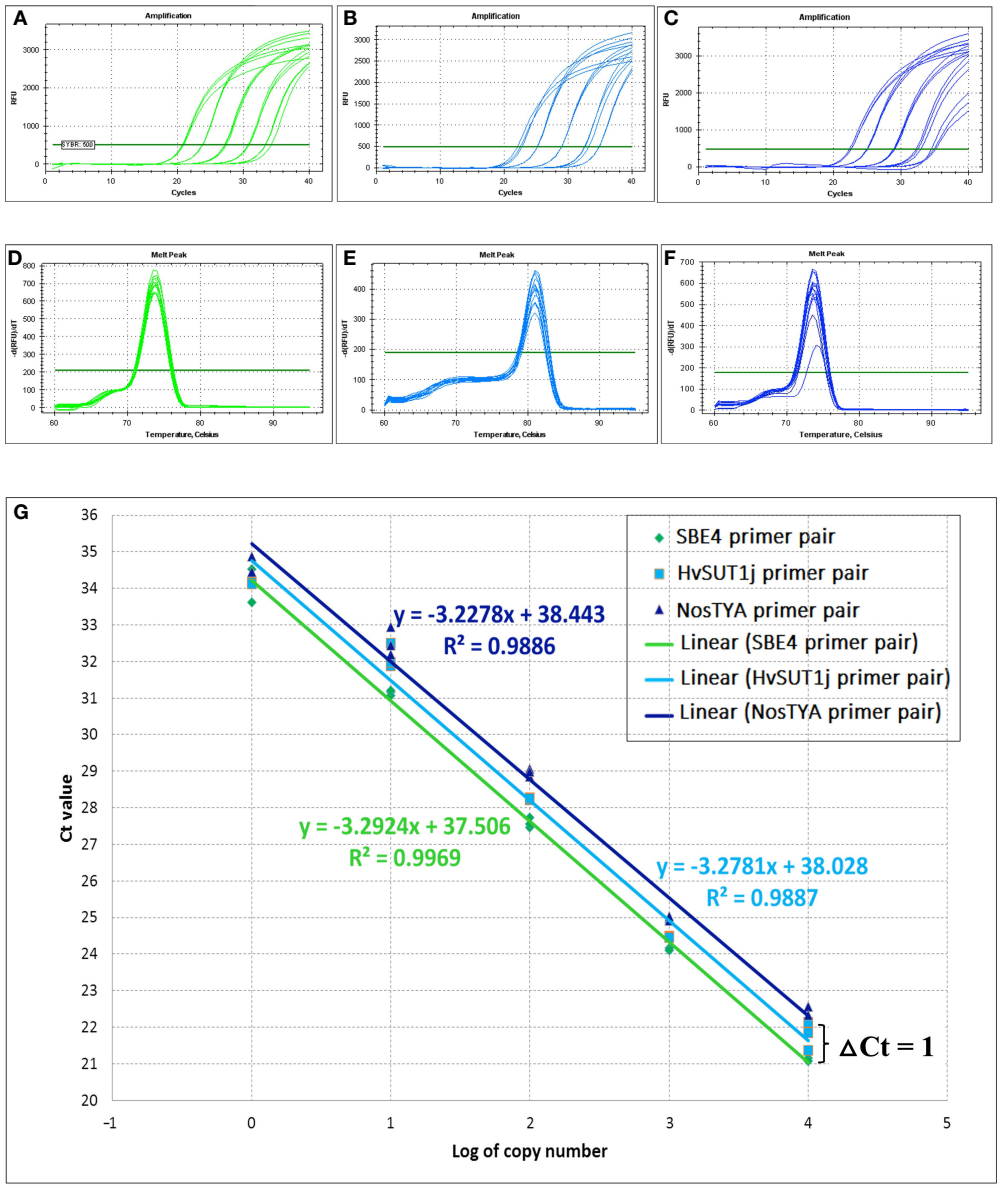
Amplification efficiencies of well-matched primer pairs used in the qPCR assay. Amplification curves and melting curves of SBE4 primer pair **(A, D)**, HvSUT1j primer pair **(B, E)** and NosTYA primer pair **(C, F)** for the qPCR assay were conducted in a CFX96™ Real-time PCR detection system (BioRAD). The fluorescent threshold was set at 500 Relative Fluorescent Units (RFU). **(G)** Comparison in amplification efficiency of SBE4, HvSUT1j and NosTYA primers. The genomic DNA from the A5.1 transgenic line with a single T-DNA insert was diluted by 10-fold serial dilution (10^0^, 10^1^, 10^2^, 10^3^, and 10^4^ copies/µl). The standard curve for each primer pair was made by plotting the average Ct value of three replicates for each dilution against the logarithm of the DNA starting quantity. The green, light blue and dark blue regression lines with their equations and R^2^ values are presented for SBE4, HvSUT1j and NosTYA primers.

To reliably determine the zygosity of transgenic lines using the qPCR protocol, the comparative 2^-ΔΔCt^ method was used for copy number estimation (Bubner and Baldwin, 2004). This method requires similar PCR efficiencies of the T-DNA amplicons and an endogenous reference gene present as two copies at a single locus in the diploid rice genome. Thus, specific and well-matched sets of primer pairs for the T-DNAs and reference genes were designed and it was verified that their amplification efficiencies were close to 100% ± 5%. Three primer pairs for two endogenous reference genes, namely the starch branching enzyme 4 (*OsSBE4*) and the sucrose phosphate synthase (*OsSPS*), five primer pairs for the *HvSUT1* gene (HvSUT1m, HvSUT1j, HvSUT1a, HvSUT1b, and HvSUT1c), and three primer pairs for the *nos* terminator (NosTY, NosTYA and NosTh), all present in the T-DNAs, were designed and screened in this study. All selected primer pairs were used in the qPCR assay with 10-fold serial dilutions of the genomic DNA of the A5.1 line (10^0^, 10^1^, 10^2^, 10^3^ and 10^4^ copies/μl) to determine their relative efficiencies (**Table 1**). Three primer pairs (SBE4, NostYA and HvSUTj) showed high specificity, generating single PCR products (as seen on the 1.5% agarose gel; **Figure S1**) and a single peak in the melt curve (**Figure 2**). Their reproducible amplifications had similar efficiencies close to 100%. The R^2^ values for SBE4, HvSUTj and NosTYA primers were 0.997, 0.989 and 0.989, respectively. It was interesting that the three regression lines derived from the qPCR assays with SBE4, HvSUTj and NosTYA primers were parallel. This indicates that the reaction efficiencies of the two primer pairs are very well-matched and comparable within the gDNA template concentration range of 10^0^ – 10^4^ copies/μl. Two well-matched sets of primer pairs (SBE4/HvSUTj and SBE4/NosTYA) were selected for further analyses.

**Table 1:** Screening for the specificity, PCR amplification efficiency, slope, and correlation coefficients (R^2^) for all primer pairs of the T-DNAs and internal reference genes.

Target gene	Primer pair’s name	Specificity (PCR product) number	Melt-curve analysis	Efficiency (%)	Slope	R^2^	Reference	
Reference genes	Rice Sucrose Phosphate Synthase (*OsSPS*)						
	SPSm	1 band	1 peak	108.5	-3.134	0.992	Mieog et al. (2013)	
	SPSj	1 band	No product					
	Rice Starch branching Enzyme (*OsSBE4*)						
	**SBE4**	**1 band**	**1 peak**	**102.8**	**-3.258**	**0.997**	Wang et al. (2015)	
T-DNAs	Barley sucrose transporter (*HvSUT1*)						
	HvSUT1m	1 band	1 peak	120.9	-2.906	0.979		
	**HvSUT1j**	**1 band**	**1 peak**	**101.9**	**-3.278**	**0.989**		
	HvSUT1a	3 bands	2 peaks	96.4	-3.411	0.987		
	HvSUT1b	3 bands	1 peak	84.1	-3.774	0.988		
	HvSUT1c	1 band	1 peak	122.3	-2.883	0.980		
	Nopaline synthase *(nos)* terminator						
	NosTY	1 band	–	**-**	**-**	**-**	Fletcher (2014)	
	**NosTYA**	**1 band**	**1 peak**	**104.1**	**-3.228**	**0.989**		
	NosTh	1 band	1 peak	88.4	-3.637	0.990		

Specificity of all primer pairs was identified by an endpoint PCR and the products were then visualized on the 1.5% agarose gel (**Figure S1**). Melt-curve analysis, amplification efficiency, slope and R^2^ were identified based on the standard curve conducted by the qPCR assay. Three well-matched primer pairs used in the qPCR assay to determine homozygous plants were highlighted in bold.

Based on a logarithmic standard curve generated from the serial dilution of the genomic DNA from the A5.1 transgenic line, an optimized qPCR assay was established to apply in the determination of homozygous and hemizygous plants, as described above. A dilution of 10^3^ or 10^2^ copies/μl was within the dynamic range of the reaction and considered as the optimal range of starting concentrations of genomic DNA template in the qPCR assay. It was noted that the dilutions with DNA concentration lower than 10^2^ copies/μl or higher than 10^3^ copies/μl had increased variation between technical replicates. Therefore, a concentration of 10^3^ copies/μl of genomic DNA was used as the working concentration in all subsequent experiments.

In 25 offspring of the A5.1 line analysed by this qPCR assay, the prediction was that there were four homozygous plants with the mean 2^-ΔΔCt^ value of 2.00 ± 0.107, 14 hemizygous plants (the mean 2^-ΔΔCt^ value of 1.01 ± 0.041) and seven null plants (the mean 2^-ΔΔCt^ value of 0.00 ± 0.008). These data, when analysed by Chi-square (χ^2^_0.05_) test for goodness of fit, showed no significant difference between the expected segregation ratio (1:2:1) and the observed ratio with a *P* value of 0.583 (**Table 2**). The results in **Table 2** showed that Ct value differences (ΔCt values) for homozygous plants were from 0.008 to 0.17 cycles between Ct values of NosTYA and SBE4, while the ΔCt values of hemizygous plants was from 0.92 to 1.20. For the set of SBE4 and HvSU1j primer pairs, the ΔCt values were more variable with around 0.41 for homozygotes and from 1.20 to 1.46 for hemizygotes (**Supplementary Table S4**). Moreover, on analysis the standard curves for the two sets of primer pairs, the reactions with NosTYA and SBE4 primer pairs had Ct values for the same dilutions differing by around 1 cycle, while reactions with the HvSUT1j and SBE4 primers had Ct values differing by approximately 0.6 cycles with the same dilutions (**Figure 2G**). This indicates that the primer set of SBE4 and NosTYA reflected the copy number of the *OsSBE4* reference gene (two copies) doubling the copy number of the T-DNA (one copy) in the diploid genome. In the case of this A5.1 line with a single T-DNA insertion, the quantitative PCR assay reliably distinguishes the zygosity of the transgene utilising the set of primer pairs (NosTYA and SBE4).

**Table 2:** Zygosity determination of T_2_ plants from the A5.1 transgenic line with a single T-DNA insert.

Plant Number	NosTYA/SBE4					Zygosity	Mean (± SD)
	Ct_NosT_ (± SD)	Ct_SBE4_ (± SD)	ΔCt	ΔΔCt	2^-ΔΔCt^		
6	**24.14** ± 0.042	**23.98** ± 0.048	0.17	-0.91	1.88	Homozygous	Ct_Nost_:24.46 ± 0.26. **2^-ΔΔCt^**: 2.00 ± 0.107
12	**24.36** ± 0.027	**24.20** ± 0.009	0.16	-0.96	1.95	Homozygous	
13	**24.68** ± 0.034	**24.67** ± 0.062	0.008	-1.09	2.13	Homozygous	
14	**24.67** ± 0.077	**24.59** ± 0.074	0.079	-1.02	2.03	Homozygous	
1	**25.29** ± 0.021	**24.21** ± 0.053	1.08	0.00	1.00	Hemizygous	Ct_Nost_:25.36 ± 0.26. **2^-ΔΔCt^**: 1.01 ± 0.041
2	**25.25** ± 0.012	**24.33** ± 0.061	0.92	-0.16	1.12	Hemizygous	
3	**25.08** ± 0.028	**24.05** ± 0.036	1.03	-0.05	1.03	Hemizygous	
5	**25.07** ± 0.041	**23.99** ± 0.059	1.07	-0.01	1.01	Hemizygous	
7	**25.05** ± 0.068	**24.03** ± 0.015	1.01	-0.07	1.05	Hemizygous	
9	**25.13** ± 0.076	**24.12** ± 0.061	1.01	-0.07	1.05	Hemizygous	
11	**25.80** ± 0.114	**24.70** ± 0.022	1.09	0.00	1.00	Hemizygous	
15	**25.48** ± 0.07	**24.29** ± 0.027	1.19	0.07	0.96	Hemizygous	
16	**25.24** ± 0.029	**24.13** ± 0.009	1.100	0.01	1.00	Hemizygous	
18	**25.29** ± 0.073	**24.16**± 0.057	1.12	0.01	1.00	Hemizygous	
19	**25.60** ± 0.059	**24.51** ± 0.021	1.099	0.00	1.00	Hemizygous	
20	**25.81** ± 0.052	**24.61** ± 0.027	1.20	0.08	0.95	Hemizygous	
23	**25.43** ± 0.048	**24.36** ± 0.026	1.070	-0.03	1.02	Hemizygous	
25	**25.49** ± 0.123	**24.40** ± 0.117	1.086	-0.01	1.01	Hemizygous	
4	**36.14** ± 2.024	**23.93** ± 0.068	12.20	11.12	0.00	Null	Ct_Nost_:35.69 ± 2.62. **2^-ΔΔCt^**: 0.00 ± 0.008
8	**32.39** ± 0.239	**24.13** ± 0.022	8.26	7.18	0.01	Null	
10	**31.59** ± 0.004	**24.92** ± 0.034	6.66	5.57	0.02	Null	
17	**36.85** ± 2.12	**24.73** ± 0.041	12.68	11.60	0.00	Null	
21	**37.91** ± 1.712	**24.58** ± 0.033	13.33	12.23	0.00	Null	
22	**36.96** ± 0.551	**24.50** ± 0.017	12.46	11.36	0.00	Null	
24	**38.02** ± 0.75	**24.31** ± 0.01	13.72	12.59	0.00	Null	
WT	**36.85** ± 0.108	**24.18** ± 0.059	12.68	11.60	0.00	WT	
		Mean _Ct value_: 24.32 ± 0.28				4:14:7	P= 0.583^ns^ χ^2^_0.05 = _1.08

Headings indicate the set of primer pairs used in the qPCR assay. ΔCt, ΔΔCt, and 2^- ΔΔCt^ were calculated based on the formula in the MATERIALS AND METHODS. The 2^- ΔΔCt^ values of homozygous plants should be double that of the hemizygous plants. In the case of the A5.1 transgenic line with single-insert T-DNA, 2^- ΔΔCt^ values of homozygous and hemizygous plants were 2 and 1, respectively. The results were the average and standard deviation (SD) of three replicates from the same plants. All null plants were confirmed to be negative by the endpoint PCR. (ns): no significant difference. WT: non-transgenic rice plant (wild type).

To verify the accuracy of zygosity identification by the qPCR assay, T_2_ plants of the A5.1 line with a single T-DNA insert were chosen for further segregation analysis. Around 100 progeny from one homozygous plant (No. 6), three hemizygous plants (No. 1, 2 and 5), and one null plant (No. 4), as predicted by the qPCR assay, were analysed for their segregation using hygromycin selection (**Supplementary Table S5**). Progeny from the plants predicted by the qPCR to be homozygous were all resistant to hygromycin, whereas progeny from the null plant were all sensitive. Segregation of the T-DNA in the progeny of hemizygous plants showed no significant difference from the expected segregation ratio (3:1 for resistant: sensitive) (**Supplementary Table S5**). The result of the hygromycin sensitivity assay thus verified the 100% accuracy of the zygosity determined for the A5.1 line as predicted by the qPCR assay.

This optimized qPCR assay using the hemizygous plant from the A5.1 line as a calibrator, was then adapted to determine the zygosity of other transgenic lines. Two transformed lines which contained two T-DNA inserts at the same locus in the rice genome (G1.4 and G3.1) were analysed to determine their zygosity by using the optimized qPCR assay. The qPCR assay identified 2^-ΔΔCt^ values of homozygous plants with four copies as 3.9 to 4.18, and 1.93-1.98 for plants containing two copies (**Table 3** and **Supplementary Data S2**). Only plants with four, two or zero copies of the T-DNA were detected in a 1:2:1 ratio, and the segregation ratios of T-DNA inserts in the G1.4 and G3.1 lines determined by the qPCR assay suggested a single insert locus. The results of the qPCR assay were therefore consistent with the Southern blot analyses. The use of the qPCR assay to confidently detect homozygous plants where two T-DNAs had integrated at the one locus is a powerful and useful application of this tool.

**Table 3:** T-DNA segregation analysis of T_1_ plants from T_0_ plants carrying single insert based on the qPCR assay.

Line	Insert number	Total plants	T_1_ segregation ^(†)^	Chi-square (χ^2^) value	p value	Percentage of homozygous plant (%)	2^-ΔΔCt^ value of homozygous plant
DC1.21	1	22	2:12:8	3.45	0.178^ns^	9.1	3.99-4.13
DC1.1	1	16	4:6:6	1.50	0.472^ns^	25.0	3.99-4.41
DC1.5	1	24	5:8:11	5.67	0.059^ns^	20.8	3.91-4.05
DC1.9	1	17	1:10:6	3.47	0.176^ns^	5.9	3.78
DC1.13	1	38	7:19:12	1.32	0.518^ns^	18.4	4.04-4.27
SC1.22	1	16	5:8:3	0.50	0.779^ns^	31.2	1.78-2.17
SC1.21	1	22	3:12:7	1.64	0.441^ns^	13.6	1.80-2.12
SC1.12	1	8	3:4:1	1.00	0.606^ns^	37.5	2.01-2.08
SC2.15	1	21	2:14:5	3.19	0.200^ns^	9.5	1.75-1.87
SC2.31	1	25	2:17:6	4.52	0.104^ns^	8.0	1.85-2.02
SC2.14	1	8	2:4:2	0.00	1.00^ns^	25.0	1.93-1.79
SC2.32	1	14	2:7:5	1.46	0.481^ns^	14.3	1.79-1.81
G1.4	2	11	2:4:5	2.45	0.293^ns^	18.1	3.96-4.01
G3.1	2	5	2:1:2	1.80	0.407^ns^	40.0	3.9-4.18
SC1.16	2	10	3: 0: 5: 0: 2	174.67	0.00*	30.0	3.79-4.18
SC1.13	2	11	1: 0: 5: 0: 5	298.84	0.00*	9.1	3.95
	Total	268			Average (%)	19.72	

The copy number of the T-DNA was estimated by the qPCR assay using the set of NostYA and SBE4 primers. χ^2^ test (χ^2^_0.05 = _5.99; dF=2) for goodness of fit was applied to compare whether there was a significant difference between the observed segregation and expected segregation at p< 0.05. (†): In the case of a single insert, the segregation of T-DNA is 1: 2: 1 for 0: 1: 2 copies, respectively. The independent segregation of two T-DNA inserts in case of two-insert events is expected to be 1:4:6:4:1 for 4:3:2:1:0 copies, respectively. The hemizygous, single-insert plant from the A5.1 line was used as a calibrator. (*): significant difference; (ns): no significant difference.

### Application of the qPCR method

Having optimized the qPCR assay, this method was successfully applied to fast-track the identification of T-DNA copy number and zygosity of transgenic Indica rice plants in the T_1_ generation. In this experiment, three populations of transgenic Indica rice lines carrying T-DNAs with a single transgene of the *HvSUT1*, the rice nicotinanamine synthase (*OsNAS2*), and both the transgenes (*HvSUT1* and *OsNAS2*) were developed and designated as SC1, SC2 and DC1 lines, respectively (**Figures 1C–E**). All the transgenic lines contained the same *nos* sequence element as the A5.1 line (**Figure 1B**), so the qPCR assay with the same primer pairs for T-DNAs and reference gene could be used without any adaptation. The hemizygous plant from the A5.1 line was used as a calibrator for all qPCR runs. To achieve the fastest route for developing a homozygous line, we could detect single-insert lines in the T_0_ generation.

**Figure 3:**
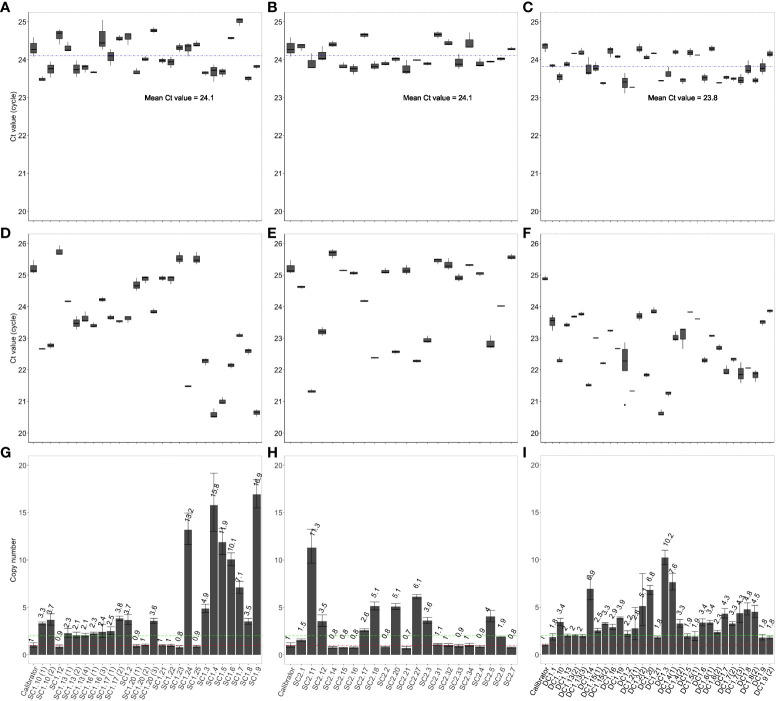
Copy number determination in T_0_ transgenic rice plants using the qPCR assay. Average Ct values of SBE4 **(A)** and NosTYA **(D)** and copy number of T-DNAs **(G)** in T_0_ transgenic rice plants carrying the SC1 T-DNA. Average Ct values of SBE4 **(B)** and NosTYA **(E)** and copy number of T-DNAs **(H)** in T_0_ transgenic rice plants carrying the SC2 T-DNA. Average Ct values of SBE4 **(C)** and NosTYA **(F)** and copy number of T-DNAs **(I)** in T_0_ transgenic rice plants carrying the DC1 T-DNA with two stacked genes. The qPCR assay was adapted without any modification. The A5.1 transgenic line was used as a calibrator. The blue lines represent the mean Ct values of the SBE4 primer pair in all SC1 and SC2 lines (24.1) and DC1 lines (23.8). Red and green lines represent 1 and 2 copies of T-DNAs, respectively.

The T-DNAs in all the T_0_ putative transgenic lines exist in a hemizygous state. For single-transgene T-DNA lines (SC1 and SC2), Ct values (~ 25) of the NosTYA primer pair (**Figures 3D, E**) were higher by approximately 1, compared to the Ct values (~ 24) of the reference SBE4 primer pair (**Figures 3A, B**). The results were comparable to the hemizygous calibrator. Based on the 2^-ΔΔCt^ value of each putative transgenic line in the T_0_ generation, the copy number of T-DNA inserts was predicted, with one indicating a hemizygous single insert, two for two hemizygous inserts, etc. The results are summarized in **Figures 3G, H**, with the putative transgenic events showing a single insertion of the T-DNAs as the most frequent, with seven of the 21 SC1 lines (33.33%) and ten of the 21 SC2 lines (47.62%). The transgenic lines likely to contain a single insertion per genome in the SC1 and SC2 lines had a 2^-ΔΔCt^ value of 0.8 - 1.0, and 0.7 - 1.1, respectively. For T_0_ transgenic lines carrying the DC1 T-DNA, Ct values of the NosTYA primer pair were similar to that of the reference SBE4 primer pair, around 24, because in this case there were two *nos* terminator regions in the T-DNA (**Figures 3C, F**). The 2^-ΔΔCt^ value was ~ 2 for single-insert lines, ~ 4 for two-insert lines, etc. The result, shown in **Figure 3I**, indicates eleven of the 30 DC1 transgenic lines (36.67%) were determined to carry a single insertion of the DC1 T-DNA with 2^-ΔΔCt^ values of 1.8 – 2.2.

The T_0_ lines carrying a single insert for the SC1, SC2 and DC1 T-DNAs were grown and used to screen for homozygous plants in the T_1_ generation. The theoretical segregation of the T-DNA in single-insert lines would be consistent with a Mendelian ratio of 1:2:1 for homozygous, hemizygous and null plants. Seeds from 13 independent transgenic events in the T_0_ generation, predicted to have a single insertion by qPCR, were grown to determine zygosity in the T_1_ generation using qPCR (**Table 3**). A total of 37 homozygous T_1_ plants were predicted from the qPCR assay; 19 homozygous plants from five independent transformation events carrying the DC1 T-DNA with the 2^-ΔΔCt^ values of 3.78 - 4.41, 11 homozygous plants from three independent SC1 transgenic lines with the 2^-ΔΔCt^ values of 1.78 - 2.17 and seven homozygous plants from four independent SC2 transgenic lines with the 2^-ΔΔCt^ values of 1.75 – 2.02. A Chi-squared (χ^2^_0.05_) analysis was used to analyse the segregation ratios of the T_1_ progenies based on the qPCR results. In all cases, there was no significant difference between the observed ratios and that expected for Mendelian segregation of a single gene. The frequencies of homozygous plants carrying the SC1 T-DNA (23.9%) were much closer to the expected frequency of 25%. There were lower frequencies in the SC2 lines (only 10.3%) and in the DC1 lines (16.2%). There was no T_1_ progeny plant from the single-insert lines with more than two copies identified by the qPCR assay. For the SC1 two-insert lines, homozygous plants were identified with 2^-ΔΔCt^ values from 4.07 - 4.14, compared to 1 for the hemizygous calibrator.

To verify the reliability and accuracy of the qPCR assay to determine zygosity and number of T-DNA insertions, 105 T_2_ progeny from eleven homozygous single-insert plants and one homozygous double-insert line (shown in **Table 3**) were analyzed for the presence of the T-DNA by end-point PCR using the specific primer pairs for the transgenes (**Supplementary Table S2**). The results (**Table 4**) indicate that all progeny from the plants predicted to be homozygous for a single T-DNA insert were positive for the presence of the T-DNA by end-point PCR, demonstrating 100% accuracy for identifying homozygous plants attained from this qPCR assay in the T_1_ generation. A similar result was also achieved for the homozygous, two-insert transgenic plants determined by the qPCR. Therefore, all materials from those homozygous plants and their progenies could be confidently used for further phenotypic, molecular, and physiological analysis.

**Table 4:** Summarizing end-point PCR analysis of T_2_ plants from the homozygous T_1_ plants.

Line	Insert number	Number of plants detected	Number of PCR positive plants	Percentage (%)
DC1.1	1	10	10	100
DC1.5	1	8	8	100
DC1.9	1	9	9	100
DC1.13	1	10	10	100
SC1.22	1	10	10	100
SC1.21	1	6	6	100
SC1.12	1	10	10	100
SC1.13	2	9	9	100
SC2.32	1	7	7	100
SC2.15	1	10	10	100
SC2.14	1	7	7	100
SC2.31	1	9	9	100
Total	–	105	105	100

All T_2_ progeny from homozygous T_1_ plants were positively confirmed for the presence of T-DNAs by endpoint PCR.

## Discussion

Ideally, identifying single-insert T_0_ transgenic lines can greatly assist in the development of homozygous T_1_ lines. However, determining homozygotes is challenging because neither end-point PCR nor Southern blotting can clearly and reliably differentiate between homozygous and hemizygous plants at this point in the experimental timeline. Therefore, genetic analysis of T_2_ progeny is required, which is time-consuming and space and labour-intensive. Alternatively, the qPCR assay can determine copy number and zygosity (Ingham et al., 2001; German et al., 2003; Bubner and Baldwin, 2004; Bubner et al., 2004; Mieog et al., 2013; Wang et al., 2015), however these studies have limitations in reliability and universality. A standardized and universal qPCR protocol to identify homozygous plants accurately, reliably, and rapidly is needed and is presented here.

By using the comparative 2^-ΔΔCt^ method, the qPCR assay was optimized to provide an accurate, reliable, and powerful tool to identify single insert T_0_ plants and thus fast track the development of homozygous transgenic lines. The comparative 2^-ΔΔCt^ method requires an endogenous reference gene and equal PCR amplification efficiencies for the reference gene and the T-DNA construct (Bubner and Baldwin, 2004). In this study, the *OsSBE4* gene was used as an endogenous reference, and the *nos* terminator for the T-DNA, were targeted to design a specific, well-matched pair of primers to achieve equal amplification efficiencies. The genomic DNA from the A5.1 transgenic line with a single T-DNA insert, as confirmed by Southern blot analysis, contains one copy of the *nos* terminator and two copies of the *OsSBE4* reference gene (Mizuno et al., 2001). The A5.1 line was used as starting DNA to analyse the PCR efficiencies of the SBE4 and NosTYA primer pairs by plotting the average Ct values for each dilution and fitting a standard curve (**Figure 2G**). These standard curves showed close to 100% amplification efficiency in each case. The difference in Ct values at each dilution between the SBE4 and NosTYA primers was consistently one cycle and parallel. This reflects the difference in copy number of the T-DNA (one copy) and the endogenous reference gene (two copies). From these results, the optimized protocol of the qPCR assay used in this study was established with standardized procedures for dynamic DNA concentration, well-matched primer pairs, real-time PCR conditions, and the 2^-ΔΔCt^ method. The qPCR assay was utilized to screen the progeny of hemizygous, single T-DNA insert lines. The results indicated 100% accuracy in determining homozygous (two copies), hemizygous (one copy), and null (zero copy) plants. Two major factors contributed to the high accuracy of the qPCR assay achieved in this study. The first is an internal reference gene with a single copy per haploid genome. Many previous reports did not determine the copy number of the reference gene (Bubner and Baldwin, 2004), and this could affect the T-DNA copy number estimate. In practice, a reference gene when present in multiple copies per haploid genome would be more complicated to amplify and estimate by real-time PCR using SYBR green. It is therefore of no surprise that many previous reports were less successful in accurately measuring T-DNA insert copy number (Bubner and Baldwin, 2004). Meanwhile, Mieog et al. (2013) and Wang et al. (2015) reported success in determining T-DNA copy number when the reference genes were present in a single copy per haploid genome. However, their Ct values of the reference genes and T-DNAs were variable in all plants with identical zygosity. In this study, the qPCR assay showed very little variation in the Ct values of the reference gene and T-DNAs. The robust and consistent results of the Ct values in every plant or sample was a direct consequence of our well-optimized and standardized protocol.

The second factor contributing to the accuracy and reliability of the assay was the Ct values of the endogenous gene (two copies) which in all the plants was consistently around 24 with a low standard deviation (0.28), and very low standard deviation (<0.14) between three replicates, as is required for copy number and zygosity determinations (Bubner et al., 2004). We conclude that the low variation in Ct values of the endogenous gene was easily achieved because of the high quality and consistent concentration of starting DNA template (10^3^ copies/μl or 0.426 ng/μl). In the previous reports in which Ct values of reference genes in samples varied significantly from 19 to 21 (Bubner and Baldwin, 2004; Wang et al., 2015), there were no standardized qPCR protocols. Furthermore, in our study, the Ct values of the *nos* terminator in the homozygous plants were all around 24, similar to the Ct values of the endogenous gene, but higher by 1 cycle in the hemizygous plants (around 25). This is more likely to reflect the copy number of the T-DNAs in the single-insert plants. Based on the Ct values of the reference gene and the T-DNA, the zygosity of single insert transgenic plants could then be accurately predicted. This level of consistency was not seen in data of the previous reports (Bubner and Baldwin, 2004; Wang et al., 2015).

Along with the high accuracy and reliability, the assay was demonstrated to be universal and repeatable for copy number and zygosity determination in transgenic rice. The assay was successfully adapted for determining multiple T-DNAs with the same *nos* terminator in two rice varieties (Indica and Japonica) at the same time and without any modifications. The NosTYA primer pair was used to detect the *nos* terminator which is a commonly used regulatory element in genetically modified of crops (Wu et al., 2014) and the SBE4 primer pair was used to amplify the endogenous reference gene, which is present as two copies per rice genome and well conserved in different rice including the most popular Indica and Japonica varieties (Mizuno et al., 2001; Wang et al., 2015). The qPCR-based methods established in the previous reports required several modifications for wider application, namely primer design for T-DNAs or reference genes, the selection of the appropriate endogenous gene, and the optimisation of the qPCR assay conditions. These modifications are crucial to achieve the level of accuracy reported here.

We demonstrated here, a fast-tracking strategy for high throughput development of single-insert homozygous T_1_ lines derived from single-insert T_0_ lines, as only two generations are required to identify at least one homozygous plant, from which 100% of the T_2_ progeny were homozygous. In addition, the qPCR assay has the potential to reduce cost in large-scale screening of homozygous plants. First, this qPCR method required a small amount of plant tissues (~100 mg) of T_1_ progeny to obtain enough genomic DNA (1.065 ng) for each qPCR reaction, thus we determined homozygous T_1_ transgenic Indica rice plant within 4 weeks after germination, thus reducing space and resources required for growing numerous hemizygous and null T_1_ progeny (75% in theory, compared to approximately 80.28% in this study). Second, the proposed protocol using SYBR Green qPCR, provides a simple and transferable assay to molecular breeding and transgenic research being much cheaper and easier to use than the TaqMan assay. Additionally, the data were unchanged when the qPCR reactions were set up in a 10 μl final volume, instead of 20 μl reactions, further reducing the experimental cost by a half. Finally, cheaper end-point PCR was used for initial screening of transgenic plants from the T_1_ population to remove all null plants. This reduced the experimental cost of the SYBR Green qPCR ReadyMix by a further 25%.

For T_0_ lines with two inserts located at different loci, multiple generations may be required to achieve single-copy homozygous plants (Mieog et al., 2013). The qPCR assay could identify two copies of the T-DNA but would be more difficult to distinguish between homozygous single insert progeny and hemizygous two-insert progeny. Although transgenic lines with a single insertion are preferred, two inserts of the T-DNA can still be useful in genetic studies. The qPCR assay was able to detect homozygous, two-insert plants containing four copies. In the case of the G1.4 and G3.1 lines containing two T-DNA inserts in a tandem inverted orientation at one locus (**Figures 1F, G**), a good correlation was found between the results of the qPCR and Southern blot assays. This result indicates that the qPCR assay was accurate and reliable and thus we successfully applied the assay to develop homozygous plants carrying the two-gene cassette (the DC1 T-DNAs) using a single reaction. In contrast, Wang et al. (2015) needed three separate reactions with three different primer pairs to detect homozygous lines stacked with three different gene cassettes at different loci in rice. The qPCR protocol used here with universal primer pairs (NosTYA) was employed to detect the homozygous lines stacked with two different genes using the same terminator sequence. This demonstrates the wide application of this qPCR assay to transgenic plant research.

Our method obtained accurate measurements of T-DNA copy number in rice that are very close to integer values. Such accuracy was very similar or even better that that achieved by the ddPCR method using a more sophisticated droplet digital PCR systems (Głowacka et al., 2016; Xu et al., 2016; Collier et al., 2017; Giraldo et al., 2019). For instance, our study reported that T-DNA copy number using the proposed qPCR method was 0.94 - 1.11 and 1.85 – 2.07 for single T-DNA copy and two T-DNA copy events, respectively. In our larger experiment, the measurements of T-DNA copy were 0.7 – 1.1 and 1.75 – 2.17, respectively. Meanwhile, Collier et al. (2017) reported 0.83 – 1.57 for single T-DNA copy, and 1.63 – 2.97 for two T-DNA copies, and Głowacka et al. (2016) showed 0.94 - 1.12 and 1.80 - 2.26 for single T-DNA copy and two T-DNA copy events, respectively. Recently only two reports, of Głowacka et al. (2016) and Xu et al. (2016), have demonstrated the successful used of the ddPCR to identify of homozygous plants. Unfortunately, in these cases no additional analysis was carried out to verify the segregation of the T-DNA in the next generation and thus the accuracy of the method could not be verified. In comparison to qPCR, the ddPCR method is more complicated than that proposed here. For example, the use of restriction digested genomic DNA containing two T-DNA copies in a tandem inverted orientation at one locus, which is relatively common in transgenic plants (Tzfira et al., 2004; De Buck et al., 2009), presents a challenge for the ddPCR method that is not the case in our method. In the study of Collier et al. (2017), one R5-24 transgenic rice line was estimated to contain a single T-DNA insert using the ddPCR method, but two T-DNA inserts using Southern blot. In the case of our study, the qPCR and Southern blot analysis of the G1.4 and G3.1 transgenic lines were consistent with a tandem inverted T-DNA insertion event. Furthermore, the ddPCR method has a much higher experimental cost and is more time-consuming than our qPCR method because the ddPCR relies on an initial restriction digestion of genomic DNA, expensive labelled probes specific to transgenes, and a costly droplet digital PCR system for T-DNA copy measurement (Cai et al., 2021). In addition, the primer pairs and labelled probes used in the ddPCR assay are specific to the T-DNA and thus would be time-consuming and expensive to optimize. Further, if multiple T-DNAs were investigated using the ddPCR method, the cost and time factors would be amplified.

To conclude, we present here an improved and efficient qPCR assay to identify homozygous transgenic plants confidently and economically. This assay is suitable for lower budget laboratories that are involved in transgenic research and could also be applied to a variety of transgenic plant species carrying T-DNAs with the same regulatory elements.

## Data availability statement

Sequence data may be found in the NCBI’s Genbank under accession numbers: the pIPKb001 vector (EU161567), the pIPKb003 vector (EU161569), *HvSUT1* (AJ272309), rice *Glb-1* promoter (AY427575), rice constitutive *Act-1* promoter (S44221.1), *OsSPS* (AP003437), *OsSBE4* (GQ150932), *Nos* terminator (MK078637.1), and rice *GluA2* (EU264103).

## Author contributions

HT and CS optimized the protocol. HT designed and performed the experiments. HT and MH contributed to plant transformation. HT completed statistical analysis of the data. PA, YS, CJ, and JCRS assisted HTT in conceiving the project. HT wrote the manuscript in consultation with PA. HT, PA, CS, YS, CJ, and JS discussed the results and contributed to the final manuscript.
