# Supplementary material for: An accurate, reliable, and universal qPCR method to identify homozygous single insert T-DNA with the example of transgenic rice
Source: Front Plant Sci. 2023 Oct 10;14:1221790. doi: 10.3389/fpls.2023.1221790 (PMC10600460; doi:10.3389/fpls.2023.1221790)
Supplement: Supplementary file 1 [file DataSheet_1.zip › Presentation 1 (27).PPTX]

## Slide 1
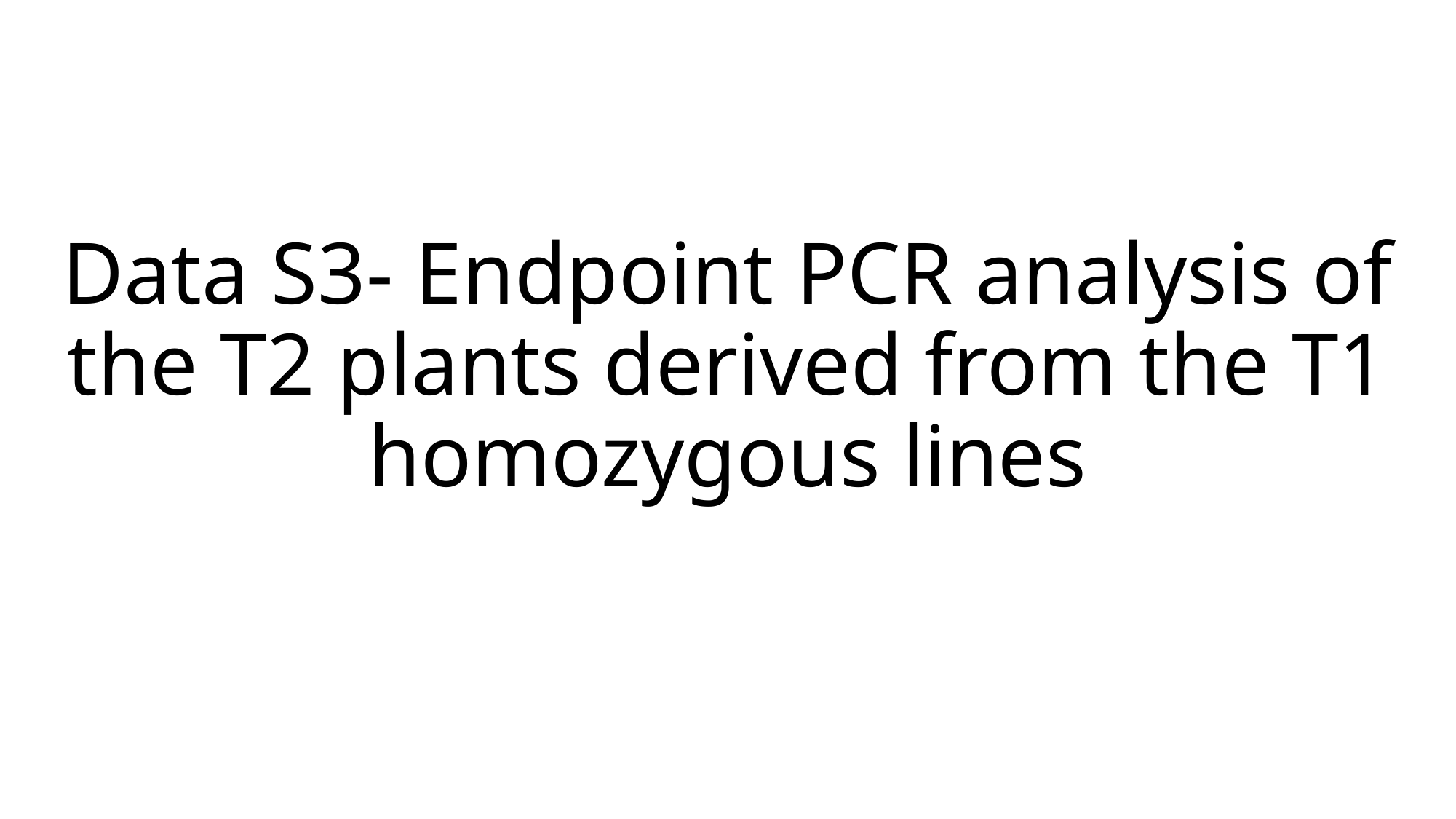

# Data S3- Endpoint PCR analysis of the T2 plants derived from the T1 homozygous lines

## Slide 2
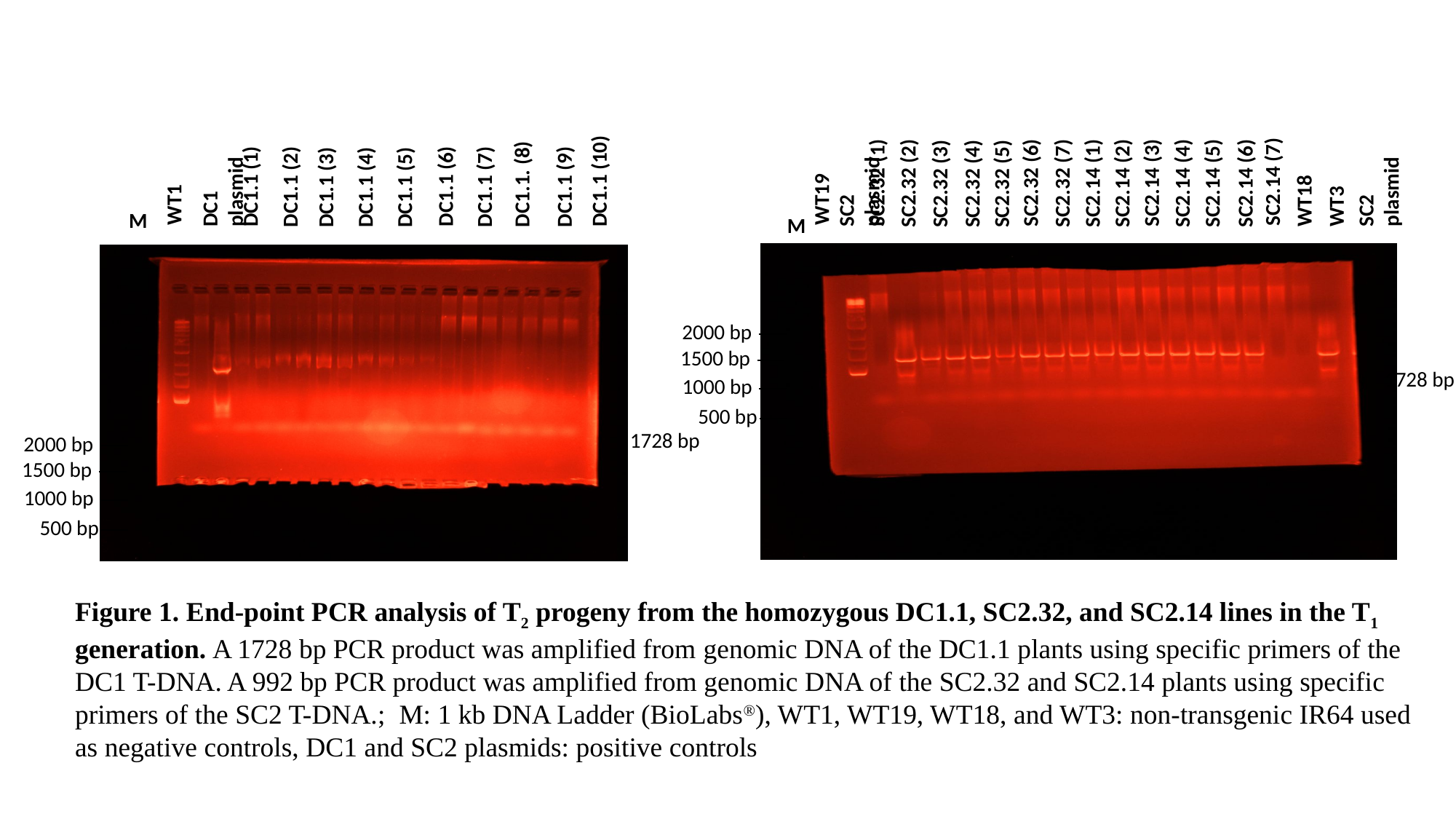

DC1.1 (1)
SC2.32 (1)
WT19
SC2.14 (7)
SC2.32 (6)
SC2.14 (3)
WT18
WT3
SC2 plasmid
SC2 plasmid
SC2.32 (2)
SC2.32 (7)
SC2.14 (1)
SC2.14 (2)
SC2.14 (4)
SC2.14 (5)
SC2.14 (6)
SC2.32 (3)
SC2.32 (4)
SC2.32 (5)
M
2000 bp
1500 bp
1000 bp
500 bp
WT1
DC1.1 (6)
DC1.1 (10)
DC1 plasmid
DC1.1 (2)
DC1.1 (7)
DC1.1. (8)
DC1.1 (9)
DC1.1 (3)
DC1.1 (4)
DC1.1 (5)
M
1728 bp
1728 bp
2000 bp
1500 bp
1000 bp
500 bp
Figure 1. End-point PCR analysis of T2 progeny from the homozygous DC1.1, SC2.32, and SC2.14 lines in the T1 generation. A 1728 bp PCR product was amplified from genomic DNA of the DC1.1 plants using specific primers of the DC1 T-DNA. A 992 bp PCR product was amplified from genomic DNA of the SC2.32 and SC2.14 plants using specific primers of the SC2 T-DNA.; M: 1 kb DNA Ladder (BioLabs®), WT1, WT19, WT18, and WT3: non-transgenic IR64 used as negative controls, DC1 and SC2 plasmids: positive controls

## Slide 3
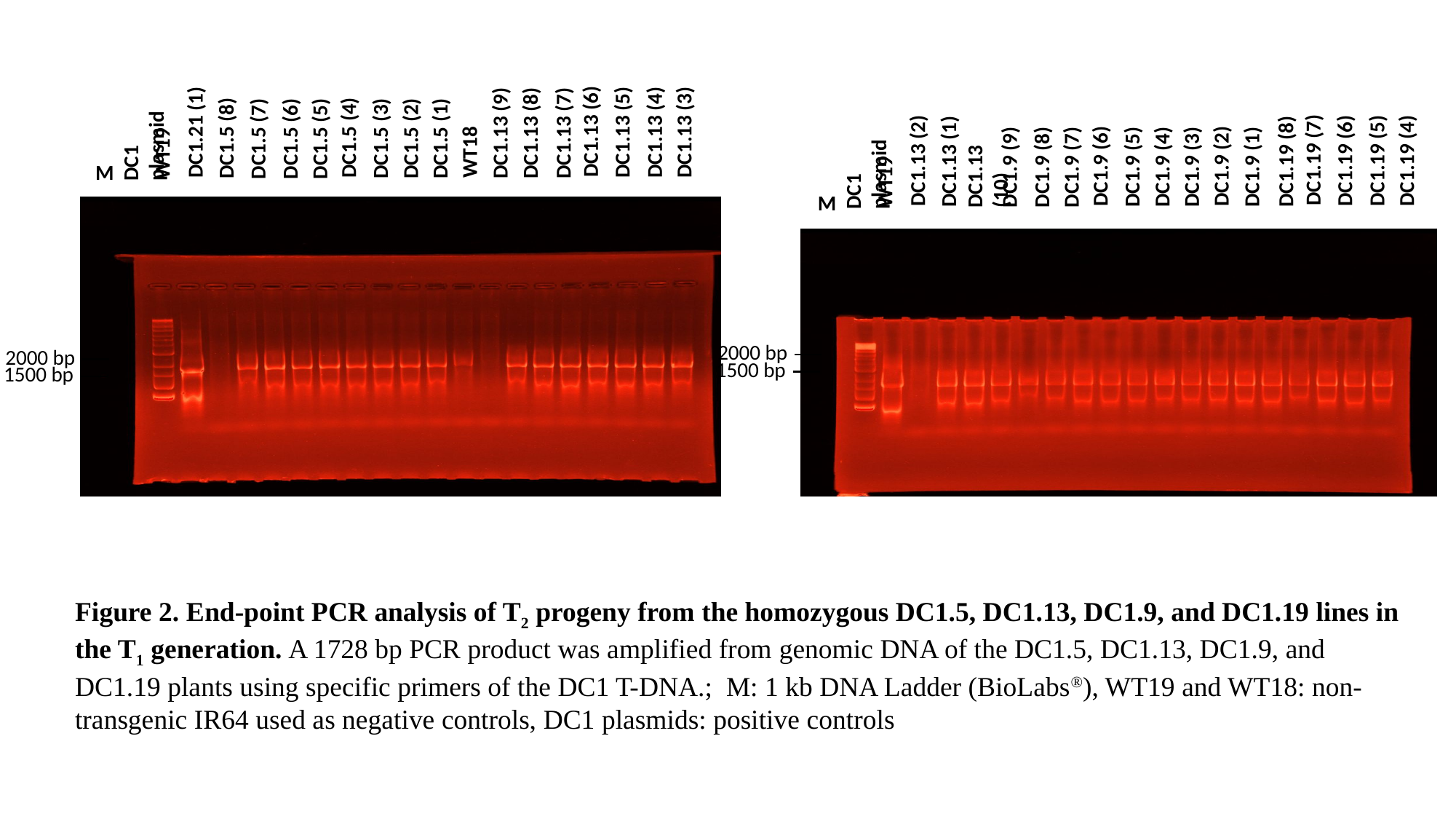

DC1.21 (1)
DC1.13 (6)
DC1.5 (4)
WT18
DC1.13 (5)
DC1.13 (4)
DC1.13 (3)
DC1.5 (8)
DC1.5 (3)
DC1.5 (2)
DC1.5 (1)
DC1.13 (9)
DC1.13 (8)
DC1.13 (7)
WT19
DC1.5 (7)
DC1.5 (6)
DC1.5 (5)
DC1 plasmid
DC1.13 (2)
DC1.19 (7)
DC1.9 (6)
DC1.9 (2)
DC1.19 (6)
DC1.19 (5)
DC1.19 (4)
DC1.13 (1)
DC1.9 (5)
DC1.9 (4)
DC1.9 (3)
DC1.9 (1)
DC1.19 (8)
WT19
DC1.13 (10)
DC1.9 (9)
DC1.9 (8)
DC1.9 (7)
DC1 plasmid
M
M
2000 bp
2000 bp
1500 bp
1500 bp
Figure 2. End-point PCR analysis of T2 progeny from the homozygous DC1.5, DC1.13, DC1.9, and DC1.19 lines in the T1 generation. A 1728 bp PCR product was amplified from genomic DNA of the DC1.5, DC1.13, DC1.9, and DC1.19 plants using specific primers of the DC1 T-DNA.; M: 1 kb DNA Ladder (BioLabs®), WT19 and WT18: non-transgenic IR64 used as negative controls, DC1 plasmids: positive controls

## Slide 4
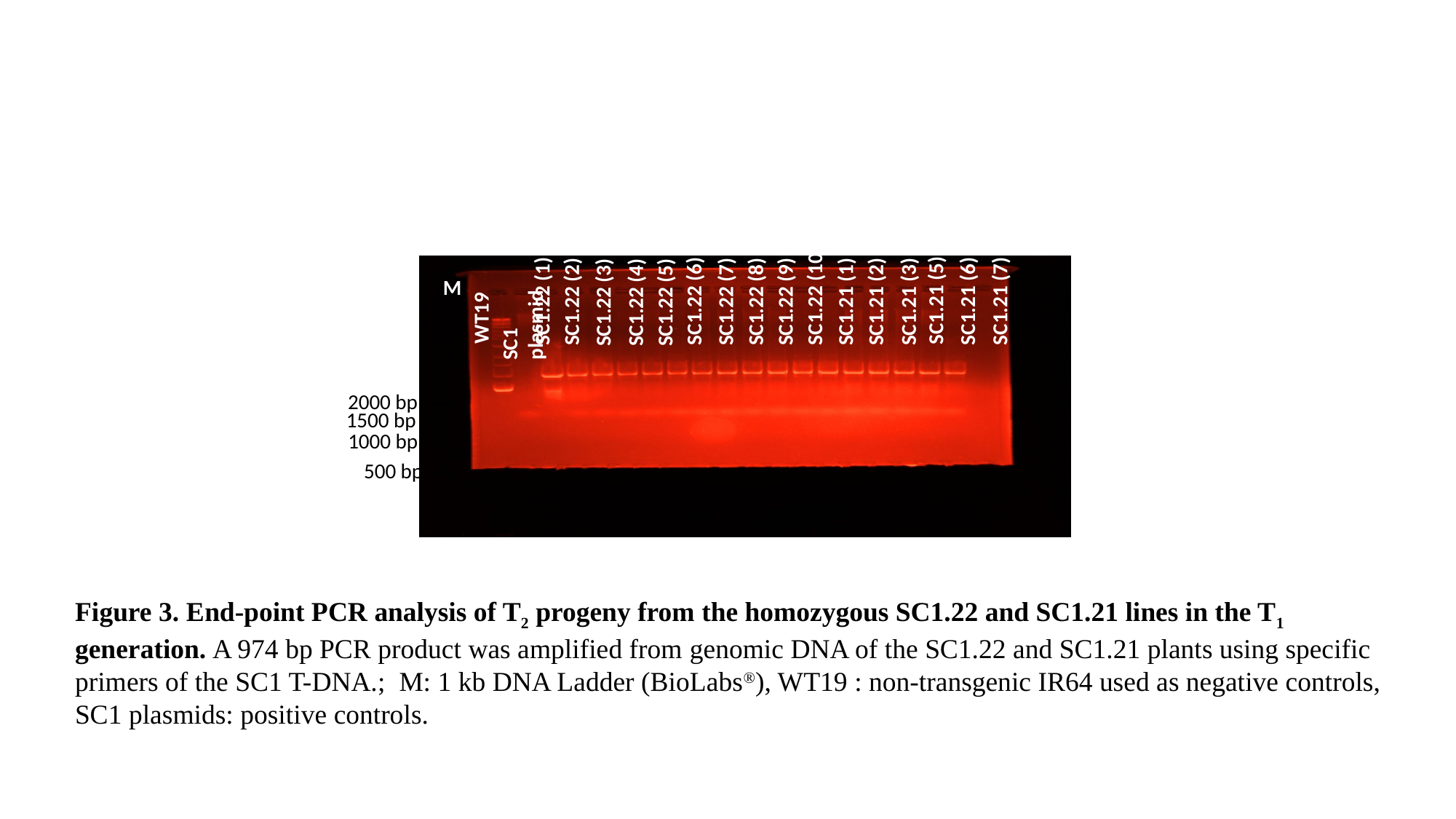

SC1.22 (1)
WT19
SC1.21 (5)
SC1.22 (6)
SC1.22 (10)
SC1.21 (6)
SC1.21 (7)
SC1.22 (2)
SC1.22 (7)
SC1.22 (8)
SC1.22 (9)
SC1.21 (1)
SC1.21 (2)
SC1.21 (3)
SC1.22 (3)
SC1.22 (4)
SC1.22 (5)
SC1 plasmid
M
2000 bp
1500 bp
1000 bp
500 bp
Figure 3. End-point PCR analysis of T2 progeny from the homozygous SC1.22 and SC1.21 lines in the T1 generation. A 974 bp PCR product was amplified from genomic DNA of the SC1.22 and SC1.21 plants using specific primers of the SC1 T-DNA.; M: 1 kb DNA Ladder (BioLabs®), WT19 : non-transgenic IR64 used as negative controls, SC1 plasmids: positive controls.

## Slide 5
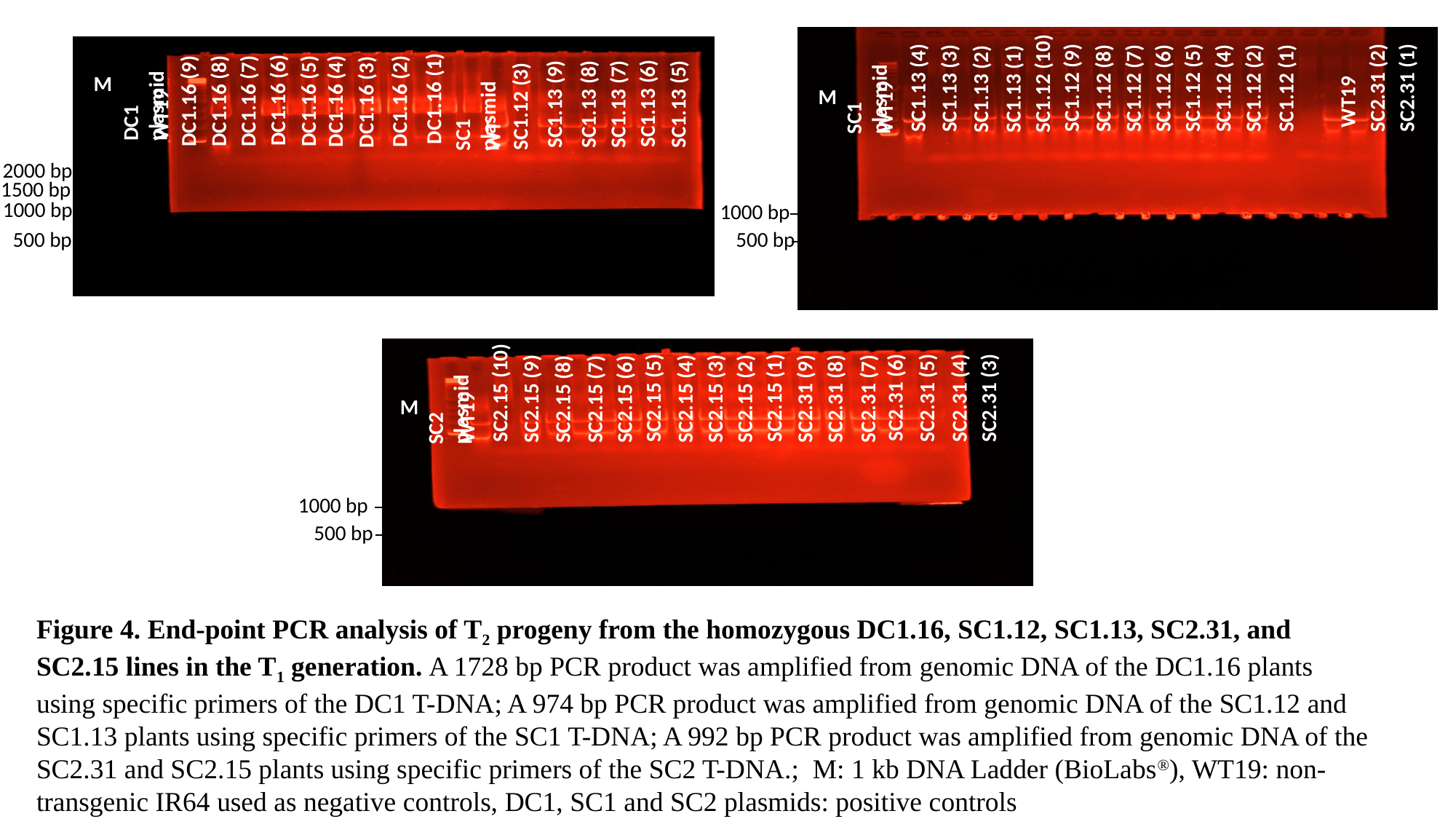

SC1.13 (4)
WT19
SC1.12 (9)
SC1.12 (5)
SC2.31 (2)
SC2.31 (1)
SC1.13 (3)
SC1.12 (8)
SC1.12 (7)
SC1.12 (6)
SC1.12 (4)
SC1.12 (2)
SC1.12 (1)
WT19
SC1.13 (2)
SC1.13 (1)
SC1.12 (10)
SC1 plasmid
DC1 plasmid
WT19
DC1.16 (1)
DC1.16 (6)
DC1.16 (9)
DC1.16 (8)
DC1.16 (7)
DC1.16 (5)
DC1.16 (4)
DC1.16 (2)
SC1.13 (6)
DC1.16 (3)
SC1.13 (8)
SC1.13 (7)
SC1.13 (5)
SC1.13 (9)
SC1.12 (3)
SC1 plasmid
WT
M
M
2000 bp
1500 bp
1000 bp
1000 bp
500 bp
500 bp
SC2.15 (1)
SC2.15 (10)
SC2.31 (6)
SC2.15 (5)
SC2.31 (5)
SC2.31 (4)
SC2.31 (3)
SC2.15 (9)
SC2.15 (4)
SC2.15 (3)
SC2.15 (2)
SC2.31 (9)
SC2.31 (8)
SC2.31 (7)
WT19
SC2.15 (8)
SC2.15 (7)
SC2.15 (6)
SC2 plasmid
M
1000 bp
500 bp
Figure 4. End-point PCR analysis of T2 progeny from the homozygous DC1.16, SC1.12, SC1.13, SC2.31, and SC2.15 lines in the T1 generation. A 1728 bp PCR product was amplified from genomic DNA of the DC1.16 plants using specific primers of the DC1 T-DNA; A 974 bp PCR product was amplified from genomic DNA of the SC1.12 and SC1.13 plants using specific primers of the SC1 T-DNA; A 992 bp PCR product was amplified from genomic DNA of the SC2.31 and SC2.15 plants using specific primers of the SC2 T-DNA.; M: 1 kb DNA Ladder (BioLabs®), WT19: non-transgenic IR64 used as negative controls, DC1, SC1 and SC2 plasmids: positive controls
