# Supplementary material for: An accurate, reliable, and universal qPCR method to identify homozygous single insert T-DNA with the example of transgenic rice
Source: Front Plant Sci. 2023 Oct 10;14:1221790. doi: 10.3389/fpls.2023.1221790 (PMC10600460; doi:10.3389/fpls.2023.1221790)
Supplement: Supplementary file 1 [file DataSheet_1.zip › Table 1 - 2023-09-25T092535.956.DOCX]

Supplementary Materials

An accurate, reliable, and universal qPCR method to identify homozygous single-insert T-DNA with the example of transgenic rice

Hai Thanh Tran*, Carly Schramm, My-my Huynh, Yuri Shavrukov, James C.R. Stangoulis, Colin L.D. Jenkins, and Peter A. Anderson*

*** Correspondence:** Hai Thanh Tran. E-mail: haitranthanhclrri@gmail.com; Peter A. Anderson: peter.anderson@flinders.edu.au.

# Supplementary Data

**Supplementary Data S1.** Ct values of transgenic lines in the T_0_ generation

**Supplementary Data S2.** Ct values and copy number calculation of the T_1_ generation.

**Supplementary Data S3**. Endpoint PCR analysis of the T_2_ plants derived from the homozygous T_1_ lines.

# Supplementary Figures and Tables

## Supplementary Figures


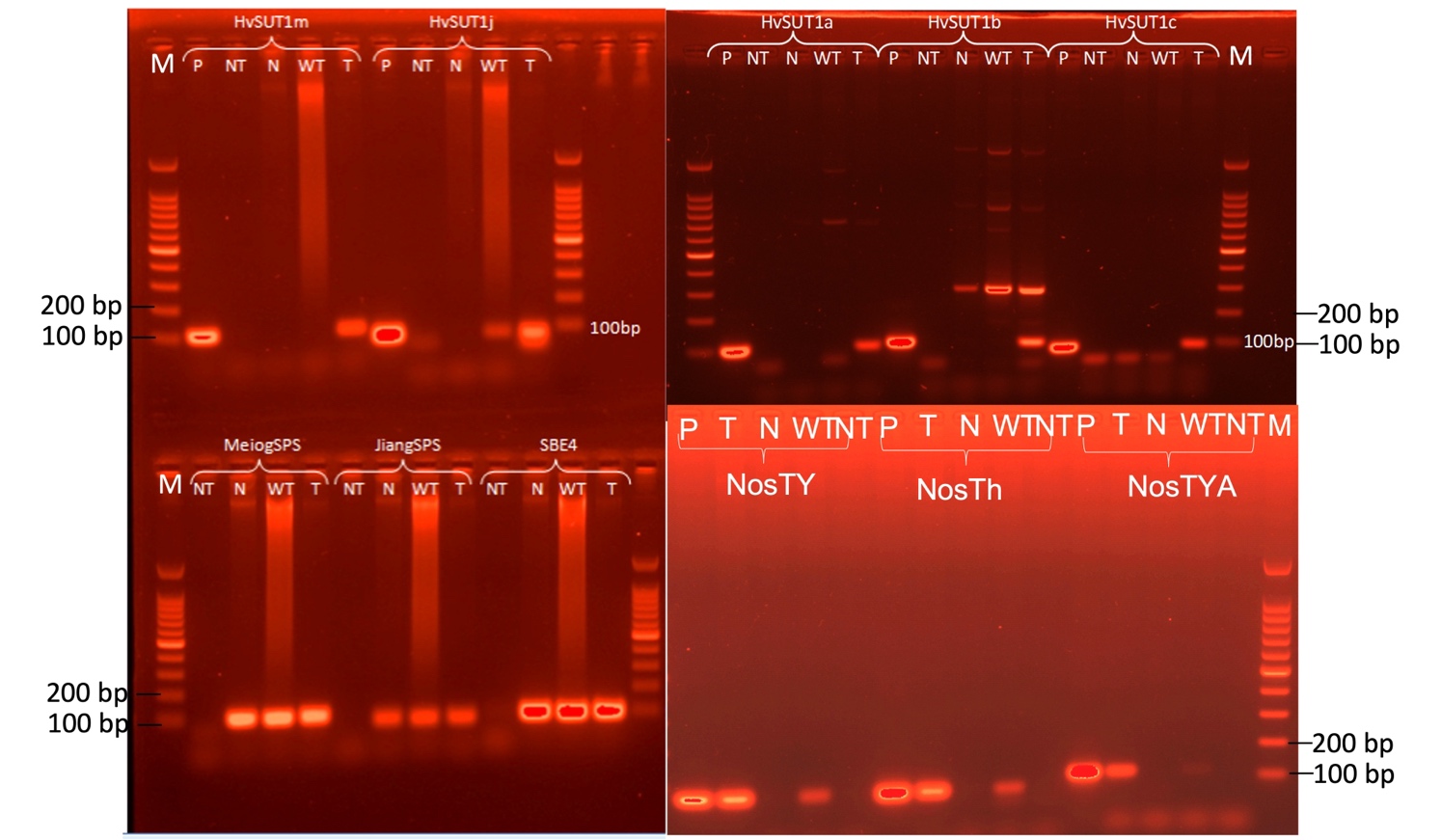


**Supplementary Figure S1.** **An initial screening for the specificity of all primer pairs using endpoint PCR**. Each primer pair was tested with no template (NT) control, a negative plant of T-DNA constructs (Null plant), non-transgenic plant or wild type (WT), and transgenic plant (T; the A5.1 line) and the pIPKb001 plasmid carrying the *Glb1:HvSUT1:NosT* (P; positive control). M: 100-bp DNA Ladder.

**
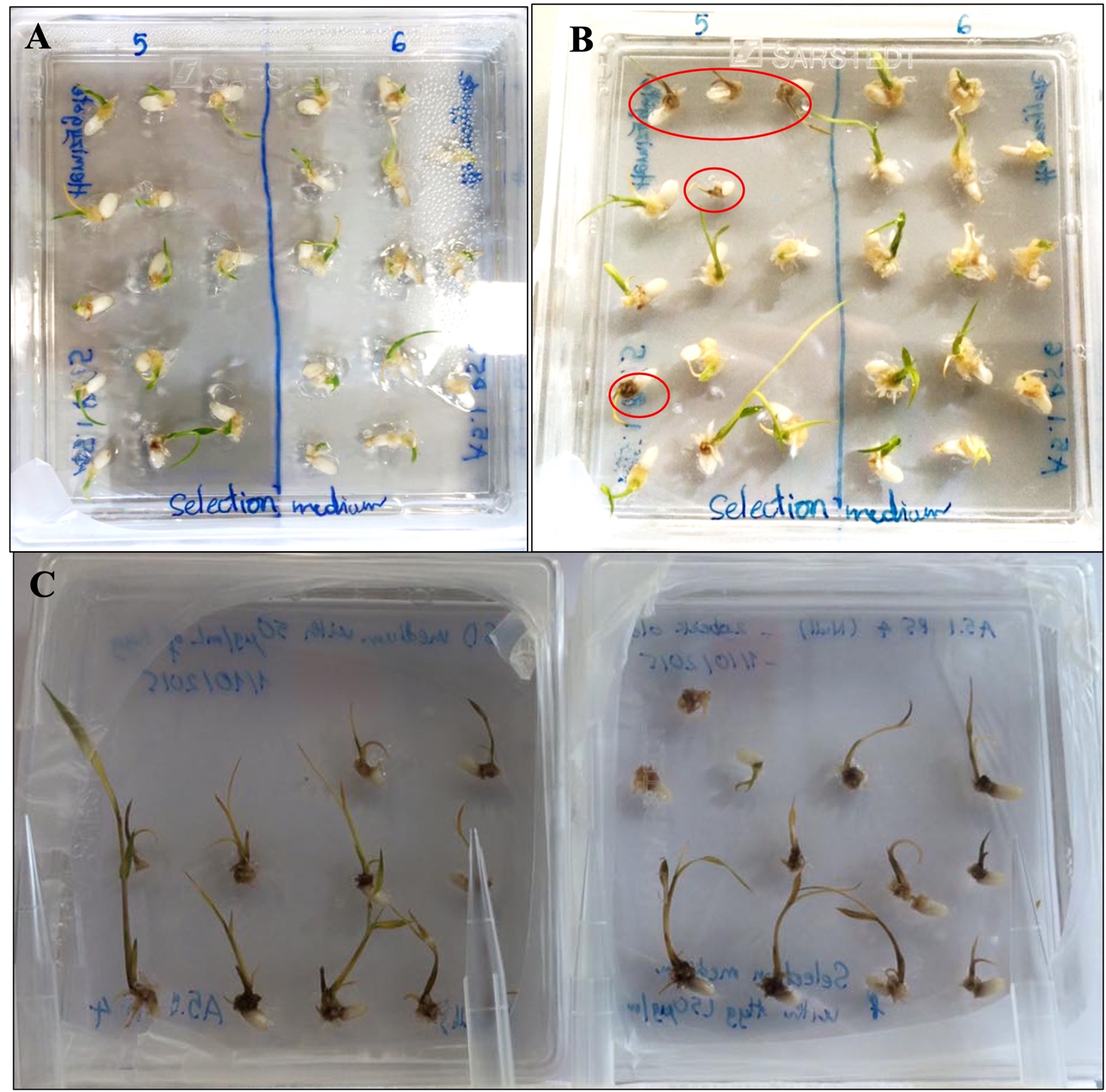
**

**Supplementary Figure S2. Segregation analysis of hygromycin phosphotransferase in the A5.1 line**. Seeds from the A5.1 transgenic line were germinated on N6D medium without hygromycin for a week before they were replaced on selection medium (N6D medium with 50 µg/ml hygromycin). **(A)** Progeny of the homozygous plant 6 and the hemizygous plant 5 on the same N6D medium with 50 µg/ml hygromycin before selection and **(B)** after selection for a week, 5/13 seedings from the hemizygous plant 5 died and 100% seedings from the homozygous plant 6 grew normally. **(C)** 100% progeny of the null plant 4 died completely on N6D medium with 50 µg/ml hygromycin after 7 days.

**
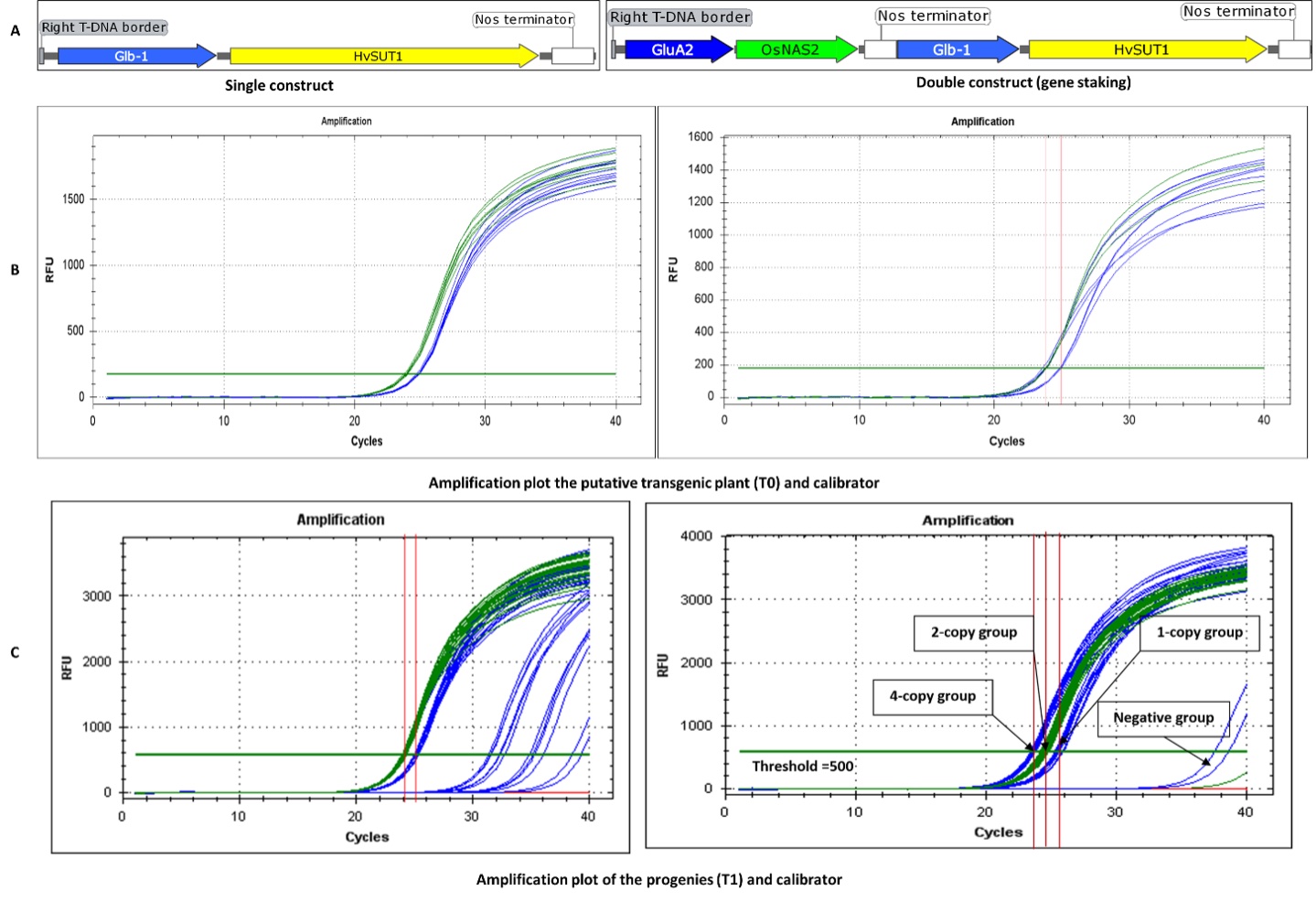
**

**Supplementary Figure S3. Performance of the qPCR assay to determine the zygosity of transgenic plants**. **A.** The SC1 T-DNA carrying a single gene cassette with a single *nos* terminator, and the DC1 T-DNA carrying double gene cassettes with two *nos* terminator regions (gene stacking); **B.** Amplification plot of a single-insert line and gene-stacking line in the T_0_ generation. **C.** Amplification plot of T_1_ offspring of the T_0_ generation. Blue and green curves represent for PCR amplifications of NosTYA and SBE4 primer pairs, and the red curve shows the no template control. The calibrator is a single-insert, hemizygous plant verified by Southern blot, the qPCR assay, and segregation testing.

## Supplementary Tables

**Supplementary Table S1.** Primer sequences for reference genes and T-DNAs used in the qPCR assay

| Primer name | Sequence (5'->3') | Direction | Target Region | PCR product size | Accession number |
| --- | --- | --- | --- | --- | --- |
| MeiogSPS | AGAGATCGACGAAAAGCGGA | Forward | *SPS* gene | 106 | AP003437 |
|  | TTTTCGGGATGATCCGAGCC | Reverse |  |  |  |
| JiangSPS | TTGCGCCCTGACGGATAT | Forward |  | 81 |  |
|  | CGGTTGATCTTTTCGGGATG | Reverse |  |  |  |
| SBE4 | GTTTTAGTTGGGTGAAAGCGGTT | Forward | *SBE4* gene | 86 | GQ150932 |
|  | CCTGTTAGTTCTTCCAATGCCCTTA | Reverse |  |  |  |
| HvSUT1m | TCTGTATGTGTGTGCCGGTC | Forward | *HvSUT1* | 107 | . AJ272309 |
|  | CGCAAGTCACAACCCAAAGG | Reverse |  |  |  |
| HvSUT1j | GAGTCACTGTATGTAGCGGA | Forward |  | 84 |  |
|  | CAAGTCACAACCCAAAGGAC | Reverse |  |  |  |
| HvSUT1a | TCACTGACTGACCTGAGCAT | Forward |  | 107 |  |
|  | CGAGAGCCACCTAGTTACGA | Reverse |  |  |  |
| HvSUT1b | CTTCAAGAACTTGCCTCCCG | Forward |  | 118 |  |
|  | GTGGTAGATCTCACGACCCA | Reverse |  |  |  |
| HvSUT1c | CTAAGGACTCCGTAGAGCAGAAT | Forward |  | 100 |  |
|  | TCCATTTTCCGCTACATACAGTGA | Reverse |  |  |  |
| NosTh | ATCATCGCAAGACCGGCAA | Forward | *Nos* Terminator | 82 | MK078637.1 |
|  | ATGACTCGAATTTCCCCGAT | Reverse |  |  |  |
| NosTY | ATCATCGCAAGACCGGCAA | Forward |  | 82 |  |
|  | GGAGCTCGAATTTCCCCGAT | Reverse |  |  |  |
| NosTYA | CGGTCTTGCGATGATTA | Forward |  | 121 |  |
|  | GTATAATTGCGGGACTCTAA | Reverse |  |  |  |

**Supplementary Table S2.** End-point PCR conditions and primers for detecting the transgene in the T-DNAs

| **Component** | **25-µl reaction** | **Final concentration** | |  |
| --- | --- | --- | --- | --- |
| MiliQ water | 13.375 |  | |  |
| 5 × Green buffer (Promega) | 5 | 1× | |  |
| 25mM MgCl_2_ | 2 | 2 mM | |  |
| 10mM dNTPs | 0.5 | 0.2 mM | |  |
| 5 µM Forward Primer (*) | 1 | 200 nM | |  |
| 5 µM Reverse Primer | 1 | 200 nM | |  |
| GoTaq® DNA polymerase (5 u/µl) | 0.125 |  | |  |
| Genomic DNA (10^5^ copies/µl) | 2 |  | |  |
| **Primers for end-point PCR** | | | | |
| **Name** | **Sequence (5’🡪3’)** | | **Tm** | |
| HvSUT1 3F | GGTTCTGGGGTTTAGCTCGT | | 64.2 | |
| NosTR | AAGACCGGCAACAGGATTC | | 64 | |
| OsNAS2-F1 | CTCTTCACCGACCTCGTCAC | | 65 | |
| OsNAS2-F2 | CAAGTGCTGCAAGATGGAGG | | 65.9 | |
| HvSUT1R1 | TGTCACTGTAGAGCCCAACG | | 64 | |
| HvSUT1 3F | GGTTCTGGGGTTTAGCTCGT | | 64.2 | |

Note: (*) *HvSUT1* 3F and NosTR used for detecting the SC1 T-DNA; [*OsNAS2*-F1](javascript:void(0)) and NosTR for detecting the SC2 T-DNA; [*OsNAS2*-F2](javascript:void(0)) and [*HvSUT1R1*](javascript:void(0)) for detecting the DC1 T-DNA.

**Supplementary Table S3.** N6D media used in segregation analyses of transgenic Japonica rice

| **Components** | **Amount** |
| --- | --- |
| Chu’s Basal Salt Mixture w/vitamins | 3.99 g |
| Casamino acid (or Casein hydrolysate | 300 mg |
| *myo*-inositol | 100 mg |
| L proline | 2.88 g |
| *Add 450 ml ddH_2_O and adjust pH 5.8 by 1N KOH*  *Add (0.4 % final volume) 4 g Gelrite*  *Top up ddH_2_O to 749 ml*  *Autoclave and then add* | |
| Sucrose stock solution (120 g/L) | 250 mL |
| 2,4-D (2 mg/ml) | 1 ml |
| Hygromycin B (50 mg/ml) | 1 ml |

**Supplementary Table S4.** Zygosity determination of T_2_ plants from the A5.1 transgenic line with a single T-DNA insert

| **Plant** | **HvSUT1j/SBE4** | | | | | **NosTYA/SBE4** | | | | | **Zygosity** |
| --- | --- | --- | --- | --- | --- | --- | --- | --- | --- | --- | --- |
|  | **Ct HvSUT1j** | **Ct SBE4** | **ΔCt** | **ΔΔCt** | **2^-ΔΔCt^** | **CtNosT** | **Ct SBE4** | **ΔCt** | **ΔΔCt** | **2^-ΔΔCt^** |  |
| 1 | **25.63** ± 0.069 | **24.16** ± 0.036 | 1.46 | 0.00 | 1.00 | **25.29** ± 0.021 | **24.21** ± 0.053 | 1.08 | 0.00 | 1.00 | Hemizygous |
| 2 | **25.43** ± 0.052 | **24.23** ± 0.020 | 1.20 | -0.26 | 1.20 | **25.25** ± 0.012 | **24.33** ± 0.061 | 0.92 | -0.16 | 1.12 | Hemizygous |
| 3 | **25.29** ± 0.019 | **24.02** ± 0.052 | 1.28 | -0.19 | 1.14 | **25.08** ± 0.028 | **24.05** ± 0.036 | 1.03 | -0.05 | 1.03 | Hemizygous |
| 4 | **34.93** ± 0.302 | **23.87** ± 0.038 | 11.06 | 9.60 | 0.00 | **36.14** ± 2.024 | **23.93** ± 0.068 | 12.20 | 11.12 | 0.00 | Null |
| 5 | **25.27** ± 0.051 | **23.90** ± 0.088 | 1.37 | -0.09 | 1.07 | **25.07** ± 0.041 | **23.99** ± 0.059 | 1.07 | -0.01 | 1.01 | Hemizygous |
| 6 | **24.38** ± 0.076 | **23.96** ± 0.079 | 0.41 | -1.05 | 2.07 | **24.14** ± 0.042 | **23.98** ± 0.048 | 0.17 | -0.91 | 1.88 | Homozygous |
| 7 | **25.35** ± 0.068 | **23.97** ± 0.027 | 1.38 | -0.08 | 1.05 | **25.05** ± 0.068 | **24.03** ± 0.015 | 1.01 | -0.07 | 1.05 | Hemizygous |
| 8 | **32.85** ± 0.430 | **24.06** ± 0.021 | 8.78 | 7.32 | 0.01 | **32.39** ± 0.239 | **24.13** ± 0.022 | 8.26 | 7.18 | 0.01 | Null |
| 9 | **25.38** ± 0.105 | **24.03** ± 0.076 | 1.35 | -0.12 | 1.08 | **25.13** ± 0.076 | **24.12** ± 0.061 | 1.01 | -0.07 | 1.05 | Hemizygous |
| WT | **33.19** ± 0.310 | **23.93** ± 0.083 | 9.26 | 7.80 | 0.00 | **36.85** ± 0.108 | **24.18** ± 0.059 | 12.68 | 11.60 | 0.00 | WT |
| A5.1 P6 2 | **33.97** ± 0.488 | **24.18** ± 0.052 | 9.79 | 8.33 | 0.00 | **31.43** ± 2.119 | **24.02** ± 0.046 | 7.41 | 6.33 | 0.01 | Null |

Notes: Headings indicate the set of primer pairs used in quantitative real-time PCR assay. ΔCt, ΔΔCt and 2^- ΔΔCt^ were calculated based on the formula in the METHODS. The 2^- ΔΔCt^ value of a homozygous plant should be double that of a hemizygous plant. In the case of a single-insert line, 2^- ΔΔCt^ value of homozygous and hemizygous plants were 2 and 1 respectively. The results were the average and standard deviation of three replications from the same plants. A5.1 P6 2 was confirmed to be negative for the transgene by endpoint PCR.

## Supplementary Methods

**Supplementary** **Method S1.** Segregation analysis of hygromycin phosphotransferase in transgenic lines

T_3_ seedlings were grown on N6D medium (**Supplementary** **Table S3**) supplemented with 50 μg/ml of hygromycin. Their viability on this selection was used to determine T-DNA segregation analyses of homozygous or hemizygous versus null plants identified by the qPCR assay. About 25 rice seeds were dehusked and sterilized with 70% ethanol for 1 min, followed by a 50% commercial bleach solution with 1-2 drops of Tween 20 for 20 min. After the sterilization step, the bleach solution was removed by rinsing thoroughly with sterile Milli-Q water (5 times). The sterile seeds were placed on N6D medium solidified with 0.4% gelrite for germination and incubated under continuous light (20-25 μmol m^-2^ s^-1^) at 28 ºC for 1-2 weeks. Healthy 1-week old seedlings were transferred to selection media with 50 μg/ml hygromycin for 1-2 weeks to determine the number of resistant and sensitive seedlings. The resistant seedlings were identified if their shoots were green, and the roots continued to grow. In contrast, sensitive seedlings were chlorotic, stunted and then died. For progenies from T_2_ plants homozygous for the T-DNA, 100% seedlings should be viable on selection plates, whereas for those homozygous for the null allele, 100% of the seedlings should be unviable. Those hemizygous for a single-insert T-DNA, should segregate in a ratio of 3 resistant: 1 sensitive. The Chi-square (χ^2^) test and randomization test of goodness-of-fit was used for comparison of observed and expected data of T-DNA segregation using R package.
