# Supplementary material for: An accurate, reliable, and universal qPCR method to identify homozygous single insert T-DNA with the example of transgenic rice
Source: Front Plant Sci. 2023 Oct 10;14:1221790. doi: 10.3389/fpls.2023.1221790 (PMC10600460; doi:10.3389/fpls.2023.1221790)
Supplement: Supplementary file 1 [file DataSheet_1.zip › Table 2 (98).DOCX]

**Table S5.** Segregation analysis of hygromycin phosphotransferase in the A5.1 line

| Plant number | Segregation analysis on hygromycin | | | P value | χ^2^ | Zygosity ^(†)^ |
| --- | --- | --- | --- | --- | --- | --- |
|  | Total | Hyg^R^ | Hyg^S^ |  |  |  |
| 1 | 29 | 20 | 9 | 0.453 | 0.563^ns^ | Hemizygous |
| 2 | 27 | 17 | 10 | 0.149 | 2.086^ns^ | Hemizygous |
| 4 | 25 | 0 | 25 | 100% |  | Null |
| 5 | 13 | 9 | 4 | 0.231 | 0.631^ns^ | Hemizygous |
| 6 | 13 | 13 | 0 | 100% |  | Homozygous |
| Total | **107** | **54** | **53** |  |  |  |

Notes: χ^2^ test (χ^2^_0.05_ = 3.841; dF=1) for goodness of fit was applied to determine if differences between the observed segregation and expected segregation (3:1 for resistant and sensitive) were significant or not. (†): Heading indicates the zygosity based on the qPCR results in **Table 2**. Hyg^R^: resistant to Hygromycin; Hyg^S^: sensitive to hygromycin. (ns): No significant difference from expected segregation ratio using χ^2^ test.
